# Factors Associated with Perceived Coercion in Adults Receiving Psychiatric Care: A Scoping Review

**DOI:** 10.3390/healthcare13151868

**Published:** 2025-07-30

**Authors:** Clara Lessard-Deschênes, Pierre Pariseau-Legault, Vincent Billé, Sophie Sergerie-Richard, Emilie Hudson, Benedetta Silva, Jean-Simon Drouin, Marie Désilets, Marie-Hélène Goulet

**Affiliations:** 1 Faculté des Sciences Infirmières, Université de Montréal, 2375, Chemin de la Côte-Ste-Catherine, Montreal, QC H3T 1A8, Canada; vincent.bille@umontreal.ca (V.B.); sophie.sergerie-richard@umontreal.ca (S.S.-R.); emilie.hudson@umontreal.ca (E.H.); jean-simon.drouin@umontreal.ca (J.-S.D.); marie-helene.goulet@umontreal.ca (M.-H.G.); 2 Centre de Recherche de l’Institut Universitaire en Santé Mentale de Montréal, Montreal, QC H1N 3V2, Canada; 3 Département des Sciences Infirmières, Université du Québec en Outaouais, Gatineau, QC J8X 3X7, Canada; pierre.pariseau-legault@uqo.ca; 4 Centre de Recherche de Montréal Sur Les Inégalités Sociales (CREMIS), Montreal, QC H2X 1K6, Canada; 5 Community Psychiatry Service, Department of Psychiatry, Lausanne University Hospital and University of Lausanne, 1011 Lausanne, Switzerland; benedetta.silva@chuv.ch; 6 Cantonal Medical Office, General Directorate for Health, Canton of Vaud Department of Health and Social Action, 1014 Lausanne, Switzerland; 7 Institut Universitaire en Santé Mentale de Montréal, Montreal, QC H1N 3M5, Canada; mdesilets.iusmm@ssss.gouv.qc.ca

**Keywords:** coercion, psychiatry, mental health, perceived coercion, formal coercion, informal coercion, factors, restrictive practices

## Abstract

Background/Objectives: Perceived coercion has been associated with significant negative outcomes, including service avoidance and psychological distress. Despite growing interest, no recent comprehensive review has mapped the full range of factors influencing this experience. This scoping review aimed to synthesize and present the state of knowledge on the factors associated with perceived coercion by adults receiving psychiatric care. Methods: Following the Joanna Briggs Institute methodology, a systematic search of five databases and grey literature was conducted for publications from 1990 to 2025 in English and French. A total of 143 sources were included and thematically analyzed. Consultation with experts and individuals with lived experience enriched the interpretation of findings. Results: Five categories of factors were identified: individual, clinical, relational, legal, and structural. Relational and legal factors were most consistently associated with perceived coercion, while individual and clinical factors showed inconsistent findings. Structural influences were underexamined but significantly shaped the experiences of the individuals receiving care. Conclusions: Perceived coercion arises from a complex dynamic of individual, relational, and systemic influences. Reducing coercion requires moving beyond individual-level factors to address structural conditions and policy frameworks. Future research should prioritize qualitative and intersectional approaches and amplify the voices of those most affected by coercive practices in psychiatric care.

## 1. Introduction

Coercion is still a central part of mental health and psychiatric care. Despite ongoing controversy and ethical debates, as well as various initiatives to reduce its use [1], the prevalence of coercion remains high [2,3,4,5,6]. In psychiatric and mental health literature, coercion is often presented as a complex concept described in three forms: formal, informal, and perceived [7]. Formal coercion refers to the use of legally regulated coercive measures such as involuntary hospitalization, seclusion, and restraints [8]. Informal coercion consists of a range of strategies predominantly used by health professionals, often aimed at promoting treatment adherence or other behaviors aligned with normative expectations [3]. Persuasion, inducement, and threats are examples of informal coercion [9]. The current review will focus on perceived coercion, which can be described as the person’s subjective, yet valid, experience of being coerced, regardless of the presence of formal or informal coercion [5].

Although less studied than other forms of coercion, perceived coercion is nevertheless commonly reported by a large number of persons receiving psychiatric care. Studies have shown that up to 74 to 80% of involuntarily hospitalized individuals, and 22 to 25% of those voluntarily admitted for a mental health issue, report perceiving coercion [5,10]. Perceived coercion has many consequences, such as an increased risk of suicide after discharge [11], avoidance of mental health services [12], and feelings of dehumanization and isolation [5]. Yet, the understanding of how this phenomenon is experienced and why it is so prevalent remains limited.

Many studies have examined which factors could be associated with perceived coercion, for example, by studying the influence of age [13], legal status [14], the quality of interactions with health professionals [15], or procedural justice [16]. However, to the best of our knowledge, no review of the literature offers a comprehensive portrait of this phenomenon. A number of literature reviews have looked at perceived coercion, as a main or secondary outcome, by exploring the impacts of seclusion and restraint [17,18], forced medication [18], the patients’ legal status [5,19,20,21,22], and the patients’ decision-making capacity [23]. We found only one systematic review, dating back to 2011, that considered other factors, such as the patients’ quality of life or their sociodemographic characteristics [20]. This review had several limitations, including the selection of studies in English only and the absence of grey literature. Furthermore, more recent studies suggest that perceived coercion may be linked to other factors such as the perception of fairness and justice during treatment, also known as procedural justice [15,16,24,25]. Considering the lack of literature reviews that take into account all the factors that may be associated with perceived coercion, a more global and recent portrait of this subject is needed.

A scoping review method was used to present the state of knowledge on the factors associated with perceived coercion by adults receiving psychiatric care. The research question that was asked is:

What factors are associated with perceived coercion by adults receiving psychiatric care?

## 2. Methods and Analysis

The Joanna Briggs Institute (JBI) methodology for scoping reviews was followed [26]. Its clear guidelines allowed the reviewers to conduct a thorough review that may be easily replicated to ensure its validity. The nine steps of the JBI methodology were followed: (1) defining the objectives, (2) developing the inclusion criteria, (3) planning the evidence searching, selection, data extraction, and presentation, (4) searching the evidence, (5) selecting the evidence, (6) extracting the evidence, (7) analyzing the evidence, (8) presenting the results, and (9) summarizing the evidence, with the addition of a 10th step of consultation with relevant collaborators to add rigor [27,28]. The consultation took place after the initial data analysis (step 7), during which preliminary results were presented, reviewed, and discussed through direct conversation and critical revision of the written document with key contributors: a person with lived experience of psychiatric care (EH) and a researcher specialized in the field of psychiatry and perceived coercion (BS). Notably, four of the authors of this review also have experience as psychiatric nurses or nurse-managers in various care settings (CLD, VB, PPL, and SSR). The Preferred reporting items for systematic review and meta-analysis extension for Scoping Reviews (PRISMA-ScR) [29] was followed (see Supplementary Table S1). The protocol was initially registered on Open Science Framework (https://osf.io/kc7gw) and consequently published [30].

### 2.1. Eligibility Criteria

In accordance with the JBI methodology for the development of a scoping review, this review applied specific eligibility criteria for the inclusion of literature based on the type of participants, concept, context, and type of evidence.

The target population was adults aged 18 years or older who are receiving or have received psychiatric care. While no upper age limit was applied, literature focusing specifically on geriatric psychiatry was excluded due to the particularities associated with this subspecialty, such as physical comorbidities and neurodegenerative disorders, which would have introduced complexity beyond the scope of this review and made it more difficult to synthesize findings in relation to general psychiatric care. Similarly, literature on intellectual disability, perinatal psychiatry, and eating disorders was excluded. In the case of eating disorders, this exclusion was due to the specific nature of this subspecialty, which often involves considerations related to physical health (e.g., medical stabilization, nutrition) that are not generalizable to psychiatric care more broadly. In contrast, subspecialties such as psychiatric rehabilitation, forensic psychiatry, community psychiatry, and addiction psychiatry were considered eligible, as they fall within the scope of general psychiatric services and typically share more comparable contexts, practices, and populations with adult psychiatric care. Addiction psychiatry was included because individuals receiving care in these settings are commonly treated within psychiatric services and subject to similar legal and clinical dynamics concerning perceived coercion.

Literature on the factors associated with perceived coercion, understood as the subjective and personal experience of coercion, was included in this review. This association could be measured quantitatively using specific scales (e.g., The MacArthur Admission Experience Survey) or explored qualitatively through participant narratives and themes related to their experience of coercion. All types of mental health care settings were included, whether inpatient, outpatient, or community based.

The types of evidence considered encompassed a broad range of literature, including but not limited to primary studies (quantitative, qualitative, and mixed methods), literature reviews (such as systematic reviews and meta-analyses), conference abstracts, guidelines, theoretical articles, and grey literature (e.g., theses). Only sources available in full text in either French or English were included.

### 2.2. Search Strategy

A search was conducted in five databases: CINAHL, MEDLINE, PUBMED, EMBASE, and PsychINFO by a librarian specialized in mental health and psychiatry (MD). Based on two main concepts derived from the research questions, “perceived coercion” and “psychiatry/mental illness”, a list of terms was generated, from which a search was conducted using descriptors and keywords (Table 1). Years of publication were limited to 1990 and onward, considering the first studies focusing specifically on perceived coercion were published after this date. A search was then conducted specifically in mental health periodicals to identify articles that might not be in the databases. A search was also conducted to identify grey literature by searching via Google, OpenGrey, university thesis sites, and government agencies (see Supplementary Table S2). The first literature search was conducted in December 2022 and was updated in January 2025 for both databases and grey literature.

### 2.3. Source of Evidence Selection

**Figure 1:**
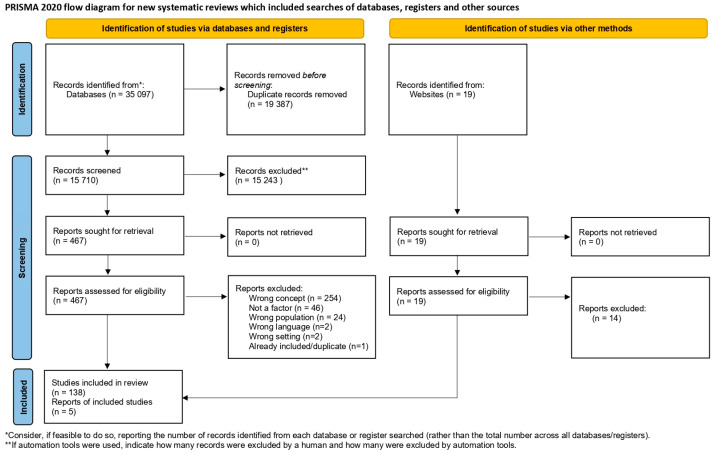
Flow diagram of the source of evidence selection process.

All citations were uploaded in Covidence software (2025). After removing duplicates, a first selection was completed based on title and abstract examination of the articles for assessment against the inclusion criteria. The selection was conducted independently by two reviewers (CLD and SSR) following a pilot test. A second selection was based on full-text examination of the literature selected in the first stage and was completed by two reviewers (CLD and EH). If any disagreements arose between the reviewers at any stage of the selection process, they were resolved through discussion to reach a final decision. The first author, who conceptualized the project, discussed any disagreements with the second reviewer and consulted her supervisor (MHG) when an additional opinion was needed. The reasons for exclusion were documented and reported in the flow diagram [29] (Figure 1).

### 2.4. Data Extraction

Data extraction was performed according to the categories proposed in the JBI methodology for scoping reviews [26] and adapted to the purpose and research questions of the present study: authors, year of publication, country of origin, purpose, population, sample size, context of care, method, type of factor assessed and its description, method of data collection used (scale, questionnaire, interview, etc.), and key findings. Using an Excel table, the first author and a research assistant independently extracted the data after reaching an inter-judge agreement during a pilot testing phase. Discussions took place throughout the extraction process to ensure that no relevant information was missed in case of uncertainty. Although we initially planned on conducting a quality assessment of the included literature, we ultimately decided against it due to the large volume of sources and the primary objective of the review, which was to map and describe the existing body of literature rather than to assess the effectiveness of interventions or make clinical recommendations.

### 2.5. Data Analysis

The extracted data were coded inductively using Excel software, with codes generated according to the specific factors presented in the literature as associated with perceived coercion. These initial codes were organized in a table to facilitate comparison and synthesis. Following this, broader categories (or themes) were developed by grouping related codes together based on similarities in content or underlying concepts. For example, codes referring to aspects such as age, gender, or education were grouped under a broader category labeled “Sociodemographic”, while codes referring to communication or therapeutic alliance were grouped under “Relational Factors”. The categorization process was iterative and informed by current literature on coercion as well as the research team’s knowledge on the subject of interest. Specifically, we sought to capture how the identified factors related to perceived coercion at different levels (individual, clinical, relational, legal, and structural). Miles et al.’s (2020) content analysis method was followed to structure this process, ensuring that the themes faithfully represented the range of factors as they emerged from the literature [31]. A detailed example of this categorization is provided in Supplementary Table S3. Reviews were considered but excluded from the results if they did not contribute new information, in order to avoid repetition of data. All reviews are presented in Table 2, but only two reviews were included and considered in the results. The preliminary results were presented to the reviewers with lived professional, academic, and personal experiences and discussed through two separate meetings. Their input was considered and incorporated in the final results.

## 3. Results

### 3.1. Characteristics of Included Literature

This scoping review includes 143 publications addressing factors associated with perceived coercion. The included publications are presented in Table 2, organized in chronological order according to their year of publication, to illustrate the evolution of the literature over time.

**Table 2:** Characteristics of included studies.

Authors and Year of Publication	Goal/Objective	Country	Care Context	Population	Sample Size	Design	Factors Evaluated	Scale Used (If Applicable)
**Rogers (1993)** [32]	The present paper is concerned with three questions: (1) To what extent is the nominal label of “voluntary” an indicator of patients entering and remaining in hospital of their own free will? (2) What was the range of perceptions, beliefs, and circumstances surrounding those who had entered the hospital as voluntary patients but felt themselves to be there under duress? (3) What differences of view, if any, exist about treatment and services between those patients who construed their status to be genuinely voluntary compared to those who did not?	UK	Inpatient	Voluntarily admitted patients	412	Mixed methods	Legal status	N/A
**Bennett et al. (1993)** [33]	This article presents a qualitative review of the transcripts of a subset of these interviews. It attends specifically to patients’ perceptions of the morality of attempts by others—primarily family members, friends, and mental health professionals—to influence them to be admitted to the hospital, and of the morality of the process by which these influence attempts resulted in admission.	USA	Inpatient	Voluntarily and involuntarily admitted patients	70	Qualitative	Inclusion, beneficent motivation, and good faith	N/A
**Lidz et al. (1995)** [34]	This article looks at the determinants of patients’ perceptions of coercion.	USA	Inpatient	Voluntarily and involuntarily admitted patients	157	Mixed methods	Procedural justice, legal status, and sociodemographic characteristics	MacArthur Admission Experience Interview (AEI)
**Nicholson et al. (1996)** [35]	The present study was therefore conducted with three goals in mind: (1) to provide additional data on the psychometric properties of the Perceived Coercion Scale and the MacArthur Admission Experience survey from which the PCS was derived; (2) to investigate the relationship between formal legal status and patients’ perceptions of the coerciveness of hospitalization; and (3) to examine the relationships between patient characteristics, especially the degree of coercion in hospital admission, and several measures of the efficacy of psychiatric hospitalization.	USA	Inpatient	All patients admitted to WSH between 1 March 1993, and 15 June 1993	123	Mixed methods	Legal status and sociodemographic characteristics	MacArthur Admission Experience Survey (AES)
**Hiday et al. (1997)** [36]	This paper attempts to develop a better understanding of coercion in psychiatric treatment by studying patient perceptions of coercion and of two closely related constructs—patients’ perceptions of negative pressures in the hospital admission process and patients’ perceptions of fair procedures in the attempts to have them hospitalized. It also examines how these constructs are influenced by sociodemographic and clinical factors.	USA	Outpatient	Involuntarily admitted patients who had been court-ordered to outpatient commitment following hospital discharge	331	Quantitative	Clinical characteristics and sociodemographic characteristics	The authors used 15 true-false items from the MacArthur Interpersonal Relations Scale to construct three dependent variables: perceived coercion, perceived negative pressures, and perceived procedural inequity
**Hoge et al. (1997)** [37]	In this paper, we report on a study designed to provide preliminary answers to these questions based on patients’ perceptions. (1) How common are “coerced voluntaries” and “uncoerced involuntaries”? (2) When are patients coerced and by whom? Are psychiatrists, other clinicians, or agents of the mental health system pressuring patients? Or are family members and friends responsible for the coercion? (3) How are patients coerced? Are patients being coerced in ways that warrant and are suitable to legal intervention?	USA	Inpatient	Voluntarily and involuntarily admitted patients	157	Mixed methods	Legal status	MacArthur Admission Experience Interview (AEI), MacArthur Perceived Coercion Scale (MPCS)
**Cascardi et al. (1997)** [38]	This study aims to evaluate patients who were court-ordered to undergo involuntary psychiatric evaluation following the expiration of an initial “emergency” detainment. Of particular interest is whether individuals who were allowed to sign themselves into the hospital as voluntary patients experienced the admission process differently from those for whom involuntary treatment petitions were initiated. The study also seeks to assess whether patients’ perceptions of coercion were more strongly influenced by interactions with community members or with hospital staff.	USA	Inpatient	Inpatients court-ordered to Crisis Stabilization Units (CSUs) for involuntary evaluation	120	Quantitative	Legal status, sociodemographic characteristics, and locus of control	MacArthur Admission Experience Interview (AEI)
**Lidz (1998)** [39]	This commentary will outline both what is currently known and the directions future research on coercion in psychiatric care must take in the coming decade to remain relevant within our evolving mental health system.	USA	N/A	N/A	N/A	Review and commentary of the literature	Relationship with staff	N/A
**Pescosolido et al. (1998)** [40]	The purpose of this study was to systematically consider the different social processes through which people come to enter psychiatric treatment by exploring the stories told by individuals making their first major contact with the mental health system.	USA	Mixed	Inpatients and outpatients making their first major contact with the mental health treatment system	109	Mixed methods	Sociodemographic characteristics and social networks	N/A
**Lidz et al. (1998)** [41]	The purpose of this study was to determine what predicts patients’ perceptions of coercion surrounding admission to a psychiatric hospital.	USA	Inpatient	Psychiatric inpatient at two university-based hospitals, recently admitted	171	Mixed methods	Legal status, sociodemographic characteristics, coercion-related behaviors or events	MacArthur Admission Experience Interview (AEI)
**Hoge et al. (1998)** [42]	In the current study, we have included patients, family (including friends and significant others), and clinicians in an attempt to understand two related questions: (1): How do family and clinician perceptions of coercion compare with the Pprceptions of patients? (2): Are the determinants of family and clinician perceptions of coercion the same as the determinants of patient perceptions of coercion?	USA	Inpatient	Data were collected from three groups: newly admitted psychiatric patients, patients’ family members or other significant others who were involved in the admission, and admitting clinicians	433	Quantitative	Legal status and sociodemographic characteristics	MacArthur Admission Experience Interview (AEI)
**Gardner et al. (1999)** [43]	The authors examine how patients changed their evaluations of psychiatric hospitalization following hospital treatment.	USA	Inpatient	Voluntarily and involuntarily admitted patients	433	Quantitative	Legal status and sociodemographic characteristics	MacArthur Perceived Coercion Scale (MPCS)
**Poulsen (1999)** [44]	This study aims to investigate differences in perceived coercion among three groups of psychiatric patients: involuntarily committed, voluntarily admitted but later detained, and purely voluntary patients, and to identify predictors of perceived coercion.	Denmark	Inpatient	Psychiatric patients admitted to five closed psychiatric wards at Aarhus University Hospital	143	Quantitative	Legal status, clinical characteristics, social functioning, coercive measures, and sociodemographic characteristics	5-item version of the Admission Experience Scale (AES), Visual Analogue Scale (VAS)
**McKenna et al. (1999)** [45]	This study attempted to determine broad views of the current mental health legislation.	New Zealand	Inpatient	Inpatients in two acute psychiatric inpatient services in Auckland, New Zealand	138	Quantitative	Previous admission to a psychiatric hospital, previously committed under mental health legislation, and sociodemographic characteristics	MacArthur Admission Experience Survey (AES)
**Lareau (2000)** [46]	This dissertation examines differences between legally mandated and non-legally mandated patients on levels of perceived coercion to enter substance abuse treatment, as well as the effect of the therapeutic alliance on altering intake levels of perceived coercion.	USA	Inpatient	Legally mandated and non-legally mandated patients entering drug treatment at two treatment sites in Philadelphia	69	Quantitative	Legal status, therapeutic alliance, and procedural justice	MacArthur Admission Experience Survey—Short Form (AES), The Survey of Treatment Entry Pressures (STEP)
**Lidz et al. (2000)** [47]	This study aims to describe who places pressures on patients to be admitted to psychiatric hospitals and to understand the sources and nature of coercive behaviors in psychiatric admissions.	USA	Inpatient	Patients admitted to psychiatric hospitals	433	Mixed methods	Sources of pressures and types of pressures	MacArthur Perceived Coercion Scale (MPCS)
**McKenna et al. (2001)** [48]	The purpose of this study was to consider patients’ perceptions of aspects of procedural justice within the context of voluntary and involuntary admission to psychiatric hospitals in New Zealand and determine which aspects may reduce patients’ perceptions of coercion.	New Zealand	Inpatient	Patients admitted to the two acute-admitting psychiatric units of Waitemata Health, Auckland, New Zealand	138	Quantitative	Procedural justice	Perceived Coercion Scale within the MacArthur Admission Experience Survey (AES)
**Olofsson & Jacobsson (2001)** [49]	This study highlights how patients narrated their experiences of being subjected to coercion and their thoughts on how coercion could be prevented in Sweden.	Sweden	Inpatient	Involuntarily admitted patients	18	Qualitative	Patients and healthcare professionals narrated their experiences of the same coercive event	N/A
**Rosen et al. (2001)** [50]	In this study, we surveyed patients enrolled in a money management program at a university-affiliated Community Mental Health Center (CMHC) in order to (1) compare the patients’ relationship to their clinicians to their relationship with their money managers and (2) explore the diverse dimensions of clients’ experience of money management.	USA	Outpatient	Outpatients enrolled in the CMHC money management program	28	Quantitative	Legal status, sociodemographic characteristics, and therapeutic alliance	N/A
**Johansson et Lundman (2002)** [51]	The study aims to gain a deeper understanding of this experience: patients who are involuntarily admitted to psychiatric care are extremely vulnerable as a consequence of the control from others and of the personal limitations due to a psychiatric disease that can influence their own control of their lives.	Sweden	Inpatient	Involuntarily admitted patients	5	Qualitative	Autonomy, perceived coercion, information received, and influence on treatment process	N/A
**Canvin et al. (2002)** [52]	The present paper presents the findings of a qualitative investigation into service users’ perceptions and experiences of living with SDOs (Supervised Discharge Orders).	UK	Outpatient	Patient living with SDOs	20	Qualitative	Perceptions and experiences of SDOs and impact on freedom	N/A
**Watts & Priebe (2002)** [53]	Assertive community treatment (ACT) is a widely propagated team approach to community mental health care that “assertively” engages a subgroup of individuals with severe mental illness who continuously disengage from mental health services. ACT condenses a dilemma that is common in psychiatry. ACT proffers social control whilst simultaneously holding therapeutic aspiration. The clients’ perspective of this dilemma was studied in interviews with 12 clients using the “grounded theory” approach.	UK	Outpatient	Assertive community treatment patients in an impoverished inner-city area of London	12	Qualitative	Deinstitutionalization challenges and integration and community opposition	N/A
**Iversen et al. (2002)** [54]	In this paper we describe perceived coercion among patients admitted to acute wards in Norway. We applied both the direct and indirect methods to measure perceived coercion. We then compared the two approaches and examined predictors for perceived coercion.	Norway	Inpatient	Voluntarily and involuntarily admitted patients in four acute wards at two Norwegian psychiatric hospitals from October 1998 through November 1999	223	Mixed methods	Sociodemographic characteristics, length of stay, and global assessment of functioning	MacArthur Admission Experience Survey (AES), Coercion Ladder (CL)
**McKenna et al. (2003)** [55]	The study aimed to explore the impact of coercion on admissions to forensic psychiatric hospitals and to test the hypothesis that admission to forensic psychiatric hospitals would be associated with significantly greater perceived coercion than that perceived by involuntary admissions to general psychiatric hospitals. A further goal was to determine which aspects of procedural justice might reduce patients’ perceptions of coercion on admission to a forensic psychiatric hospital.	New Zealand	Inpatient	Patients admitted to forensic psychiatric hospitals and involuntary admissions to general psychiatric hospitals in New Zealand	138	Quantitative	Negative pressures, procedural justice, and emotional responses to the admission process	Perceived Coercion Scale within the MacArthur Admission Experience Survey (AES)
**Elbogen et al. (2003)** [56]	The study aimed to address these questions concerning multiple forms of leverage in treatment and to take preliminary steps toward exploring the combined effects of outpatient commitment (OPC) and representative payees on perceived coercion and treatment adherence.	USA	Outpatient	Patients who had been involuntarily admitted to 1 of 4 hospitals and who were awaiting discharge on outpatient commitment to 1 of 9 counties in north-central North Carolina	258	Quantitative	Outpatient commitment	MacArthur Perceived Coercion Scale (MPCS)
**Swanson et al. (2003)** [57]	No study to date has directly examined whether legally mandated treatment in the community significantly affects quality of life one way or the other. This paper addresses that question empirically.	USA	Outpatient	Involuntarily hospitalized patients who had been ordered to undergo a period of OPC (involuntary outpatient commitment) upon discharge	262	Quantitative	Patients were randomly assigned to be released or continue under outpatient commitment in the community after hospital discharge and were followed for one year. Quality of life, treatment characteristics, and clinical outcomes	MacArthur Admission Experience Survey (AES)
**Sørgaard (2004)** [58]	This article presents the results of an intervention study aimed at reducing the level of patient-perceived coercion in two acute wards at a psychiatric hospital in Northern Norway. The interventions consisted of procedures aimed at including the patients in the processes of formulating their treatment plan and in a continuous evaluation of their stay.	Norway	Inpatient	Inpatients in an acute psychiatric ward	190	Quantitative	Coercive measures and patronizing attitudes and behaviors	Coercion Ladder (CL)
**Taborda et al. (2004)** [59]	The main objective of the present study was the assessment of perceptions of coercion among psychiatric and nonpsychiatric (surgical and medical) patients after admission.	Brazil	Inpatient	Comparison between psychiatric patients and medical/surgical patients	205	Quantitative	Legal status and sociodemographic characteristics	MacArthur Admission Experience Survey (AES)
**Bonsack (2005)** [60]	The study aimed to assess the subjective perception of psychiatric admission by patients while still in hospital.	Switzerland	Inpatient	Patient admitted to adult psychiatric care	87	Quantitative	Legal status and sociodemographic characteristics	N/A
**Van Dorn et al. (2005)** [61]	Specifically, the current paper explores the following four interrelated questions: (1) Do persons with serious mental illness think leveraged treatment is fair and effective? (2) What are the characteristics of persons who think that leveraged treatment is fair or unfair, effective or not effective? (3) How are fairness and effectiveness related? (4) How do perceived barriers to care relate to the perceived fairness of leveraged treatment?	USA	Outpatient	Outpatients from publicly funded mental health treatment programs	1011	Quantitative	Sociodemographic characteristics, social environment, clinical, and psychological and behavioral profile	N/A
**Rose et al. (2005)** [62]	Review of the literature. This study aimed to review patients’ views on issues of information, consent, and perceived coercion about electroconvulsive therapy (ECT).	UK	Inpatient	Patients given ECT	17	Review of the literature	Perceived coercion information received about ECT	N/A
**Larsson-Kronberg et al. (2005)** [63]	The study aimed to explore the experiences of coercion among individuals undergoing assessment and treatment for substance use disorders under Swedish legislation (LVM) and to understand their perspectives on the entire process from assessment to aftercare.	Sweden	Mixed	Individuals undergoing assessment or with previous experience of assessment and involuntary care under the Swedish Care of Addicts in Certain Cases Act (LVM)	74	Quantitative	Contact with healthcare professionals, opportunities to express opinions, emotional reactions to coercive measures, substance use patterns, and treatment satisfaction	Uppsala questionnaire on coercive measures
**Bindman et al. (2005)** [64]	This study aimed to investigate predictors of perceived coercion in subjects admitted to psychiatric hospitals in the UK and to test the hypothesis that high perceived coercion at admission predicts poor engagement with community follow-up.	UK	Inpatient	Patients admitted to psychiatric hospitals	100	Quantitative	Sociodemographic characteristics, previous contact with services, and objectively coercive aspects of the admission	MacArthur Admission Experience Survey (AES)
**McKenna et al. (2006)** [65]	The aim of this study is to determine the level of coercion perceived by those under outpatient commitment in New Zealand. Emphasis is given to consideration of the presence of ambivalence and the role of processes of interaction, including procedural justice, in relation to patients’ perceptions of coercion.	New Zealand	Inpatient	Involuntary outpatient within the first year of their community treatment order presenting for their statutory clinical review	138	Quantitative	Sociodemographic characteristics, previous contact with services, and clinical characteristics	MacArthur Admission Experience Survey (AES)
**Swartz et al. (2006)** [66]	This study examined lifetime use rates and correlates of outpatient commitment or related civil court–ordered outpatient treatment in five U.S. communities.	USA	Outpatient	Outpatients from five outpatient clinics affiliated with community mental health centers located in Chicago; Durham, North Carolina; San Francisco; Tampa; and Worcester, Massachusetts	1011	Quantitative	Sociodemographic characteristics, treatment compliance, clinical characteristics, global assessment of functioning, previous contact with services, and treatment satisfaction	MacArthur Perceived Coercion Scale (MPCS)
**Kjellin et al. (2006)** [67]	The objectives of this article are to compare levels of perceived coercion among committed and voluntary patients at admission to psychiatric inpatient care across the five Nordic countries and across centers within countries and to analyze differences in perceived coercion in terms of legal prerequisites and differences in clinical practice.	Switzerland	Inpatient	Voluntarily and involuntarily admitted patients from twelve psychiatric hospitals	920	Quantitative	Sociodemographic characteristics, clinical characteristics, global assessment of functioning, and previous contact with services	MacArthur Perceived Coercion Scale (MPCS)
**Appelbaum & Redlich (2006)** [68]	This study explores aspects of the population subject to financial leverage, including modeling the predictors of leverage and examining the relationships among financial leverage, treatment compliance, and attitudes toward both treatment and the use of leverage. The goal is to better inform discussions about the legitimate extent of leverage on persons with mental disabilities and the procedures that should regulate these practices.	USA	Outpatient	Outpatients from publicly funded programs were sampled from each of five sites: Chicago, Illinois; Durham, North Carolina; San Francisco, California; Tampa, Florida; and Worcester, Massachusetts	200	Quantitative	Treatment compliance, financial leverage, and treatment satisfaction	MacArthur Perceived Coercion Scale (MPCS)
**McKenna et al. (2006)** [65]	This thorough literature search on “coercion” and “civil commitment” aimed to outline best practice management strategies for nurses during the clinical application of civil commitment of mentally ill persons.	New Zealand	Mixed	Patients admitted to acute mental health services, acute forensic mental health services, and community mental health services in New Zealand	N/A	Review of the literature	Degree of restriction associated with the service involved, pattern of communication, and procedural justice	N/A
**Castille et al. (2007)** [69]	understand why, given the objective difference in the use of coercion, there was no difference in the subjective perception of coercion.	USA	Outpatient	patients discharge from psychiatric hospital on court order or not	20	Mixed methods	Court order vs. no court order after hospital discharge relationship with case manager	Not specified
**Katsakou & Priebe (2007)** [70]	This study aimed to explore psychiatric patients’ experiences of involuntary admission and treatment by reviewing qualitative studies.	UK	Inpatient	Involuntarily admitted patients in acute general psychiatric wards	5	Review of qualitative studies	Autonomy, quality of care, and emotional impact of involuntary treatment	N/A
**Renberg et al. (2007)** [71]	This study aimed to investigate determinants for perceived coercion during the admission process among voluntarily and involuntarily admitted psychiatric patients, with special focus on sex-specific patterns.	Sweden	Inpatient	Voluntarily and involuntarily admitted psychiatric patients	282	Quantitative	Legal status and sociodemographic characteristics	MacArthur Perceived Coercion Scale (MPCS), Coercion Ladder (CL)
**Sørgaard (2007)** [72]	The purpose of this article is to analyze the differences in experienced coercion, patient involvement, and user satisfaction in three groups of patients: voluntary admitted, committed, and a group where the admission was a result of joint decisions between themselves and others.	Norway	Inpatient	Patients in three closed acute wards at the psychiatric department of Nordland Hospital located in the city of Bodø in rural Northern Norway	189	Mixed methods	Sociodemographic characteristics, autonomy, clinical characteristics, coercive measures, previous contact with services, and treatment satisfaction	Coercion Ladder (CL)
**Davidson & Campbell (2007)** [73]	This paper begins by exploring the literature on coercion and mental health practice and, in doing so, highlights arguments about the relative effectiveness of strategies and ethical dilemmas that are prevalent in this field. The paper concludes with recommendations to develop ways in which practitioners might be better prepared to work within the context of coercive policy and law.	Ireland	Inpatient	The groups consisted of the clients and their respective keyworkers: an Assertive Outreach (AO) group and a Community Mental Health Team (CMHT) group	157	Quantitative	Sociodemographic characteristics and clinical characteristics	MacArthur Perceived Coercion Scale (MPCS)
**Zervakis et al. (2007)** [74]	This paper examines the association between past involuntary commitment and current perceptions of coercion in a sample of 205 voluntarily hospitalized veterans with severe mental illness.	USA	Inpatient	Voluntarily hospitalized veterans with severe mental illness	205	Quantitative	Sociodemographic characteristics, clinical characteristics, global assessment of functioning, legal status, treatment history, coercive measures, alcohol and drug use, insight into illness severity, self-rated health, and social support	MacArthur Admission Experience Survey (AES)
**Link et al. (2008)** [75]	This study aimed to address the divergent claims of the coercion to beneficial treatment perspective and the coercion to stigma perspective using a longitudinal study of outpatient commitment among individuals with severe mental illnesses.	USA	Outpatient	Individuals between the ages of 18 and 65, ascertained in treatment facilities in the Bronx and Queens, New York City	184	Quantitative	Previous involuntary inpatient hospitalizations, assignment to mandated outpatient treatment (AOT), and perceptions of being coerced into treatment	MacArthur Perceived Coercion Scale (MPCS)
**Johansson et al. (2009)** [76]	This study elucidates the meaning care has to patients on a locked acute psychiatric ward.	Sweden	Inpatient	Patients admitted voluntarily or involuntarily to the psychiatric department of a general hospital in Western Sweden	10	Qualitative	Sociodemographic characteristics, clinical characteristics, legal status, and previous contact with services	N/A
**Stanhope et al. (2009)** [77]	This exploratory study examined the extent to which social interaction between consumers and their case managers is related to the treatment experience from the perspective of consumers. The study addressed the following questions: (1) What factors are associated with perceived coercion by the consumer? (2) To what extent are perceived coercion, the consumer-provider relationship, and consumer and service contact characteristics associated with consumers’ evaluation of a service contact?	USA	Outpatient	Consumers of the Housing First program	80	Quantitative	Sociodemographic characteristics, housing status, and service contact characteristics	N/A
**Bennewith et al. (2010)** [78]	This study aimed to assess whether adult Black and minority ethnic (BME) patients detained for involuntary psychiatric treatment experienced more coercion than similar White patients.	UK	Inpatient	Patients who had been admitted under Sections 2, 3, or 4 of the Mental Health Act 1983 in the UK or who became involuntary patients within a week of admission	778	Quantitative	Sociodemographic characteristics and ethnicity	Coercion Ladder (CL)
**Kim et al. (2010)** [79]	This study aimed to investigate variables that influence psychiatric patients’ experience of coercion and the effects of the coercion on the therapeutic relationship.	South Korea	Inpatient	Psychiatric patient	279	Quantitative	Sociodemographic characteristics, clinical characteristics, procedural justice, and coercive measures	N/A
**Fu et al. (2010)** [80]	Admission experience has been shown to be related to insight and treatment adherence. This study evaluates the clinical correlates of the Chinese Admission Experience Survey (C-AES).	Hong Kong	Inpatient	Inpatients with schizophrenia	40	Quantitative	Clinical characteristics and treatment compliance	MacArthur Admission Experience Survey (Chinese version)
**Phelan et al. (2010)** [81]	This study aimed to evaluate the effectiveness and outcomes of New York State’s outpatient commitment program, focusing on psychiatric outcomes, quality of life, perceived coercion, and stigma.	USA	Outpatient	Individuals recently mandated to outpatient commitment and individuals recently discharged from psychiatric hospitals	184	Quantitative	History of involuntary commitment and number of involuntary hospitalizations	N/A
**Latimer et al. (2010)** [82]	This study tested the hypotheses that negative, but not positive, pressures would be associated with higher perceived coercion, and that procedural justice (which we relabeled “client-centredness” for reasons described below) would be associated with lower perceived coercion. Finally, it assessed whether clinical variables were correlated with negative pressures, with client-centeredness, and with perceived coercion.	Canada	Outpatient	Assertive community treatment patients	38	Quantitative	Procedural justice, negative pressures, and sociodemographic characteristics	MacArthur Admission Experience Interview (AEI)
**Thøgersen et al. (2010)** [83]	This study aimed to explore views on—and perceptions of—coercion of patients in Danish assertive community teams.	Denmark	Outpatient	Assertive community treatment patients	6	Qualitative	Influence on treatment process, autonomy, and privacy	N/A
**Patel et al. (2010)** [84]	In this study, we investigated patients’ perspectives of coercion for both depot and oral antipsychotic treatment using a quantitative instrument. For the purposes of this study, “coercion” was defined as that perceived by the patient and did not refer to legal detention status. The null hypothesis was that reported levels of coercion would not differ according to current antipsychotic formulation (depot versus oral tablets).	UK	Outpatient	Voluntary outpatients on maintenance medication	72	Mixed methods	Sociodemographic characteristics and clinical characteristics	MacArthur Admission Experience Survey (AES)
**Jaeger & Rossler (2010)** [85]	This study aimed at investigating the factors influencing psychiatric patients’ subjective measures of perceived coercion, fairness, and effectiveness. We hypothesized that patients with experience of leverage and/or coerced voluntarism were more likely to perceive coercion and would feel they were treated with less fairness and would in consequence evaluate their treatment as less effective. An additional aim was to investigate the influence of insight into illness and socio-demographic and clinical factors on these subjective measures.	Switzerland	Mixed	Inpatients and outpatients at the Department of General and Social Psychiatry, University of Zurich	187	Mixed methods	Sociodemographic characteristics, clinical characteristics, global assessment of functioning, and treatment compliance	Modified version of the MacArthur Admission Experience Survey (AES)
**Howard (2010)** [86]	This study aimed to examine the effectiveness and cost-effectiveness of women’s crisis houses by first examining the feasibility of a pilot patient-preference randomized controlled trial (PP–RCT) design.	UK	Mixed	Women requiring voluntary admission who could be admitted to a psychiatric inpatient ward or women’s crisis house	103	Quantitative	Sociodemographic characteristics, clinical characteristics, global assessment of functioning, quality of life, and satisfaction with care	MacArthur Perceived Coercion Scale (MPCS)
**Daffern et al. (2010)** [87]	The aim of the current study is to examine perceptions of coercion and interpersonal style in patients with personality disorder and to examine the relationship between these and subsequent aggression and self-harm during hospitalization. More specifically, do patients with particular interpersonal styles (i.e., hostile and dominant) experience greater coercion, and does this result in them being more likely to act out their frustrations with aggression and self-harm?	Australia	Inpatient	Patients detained under the Mental Health Act (1983) with a legal classification of psychopathic disorder, treated in the Personality Disorder Service at Rampton Hospital	39	Mixed methods	Sociodemographic characteristics and clinical characteristics	MacArthur Admission Experience Survey (AES)
**Galon & Wineman (2010)** [88]	The primary purpose of this article is to review current information and research related to coercion and the associated concept of procedural justice in mental health treatment and to discuss the implications of this knowledge for nursing practice. A secondary purpose is to spur thought and comment within the psychiatric nursing community on forms of coercive treatment, particularly OPC.	USA	Outpatient	N/A	N/A	Review and discussion of the literature	Procedural justice, outpatient commitment, and coercive measures	N/A
**Newton-Howes et al. (2011)** [20]	This study systematically examined the empirical literature on the themes and correlates of coercion as defined by the subjective experience of patients in psychiatric care.	New Zealand	Inpatient	Articles reported on patients in secondary psychiatric care whose treatment was being managed by a consultant psychiatrist	27	Systematic review	Sociodemographic characteristics, clinical characteristics, global assessment of functioning, quality of life, and influence on treatment process	N/A
**Katsakou et al. (2011)** [89]	The present study aimed (a) to investigate whether specific sociodemographic and clinical characteristics are associated with perceptions of coercion at admission among legally voluntary patients, (b) to examine whether voluntary patients who feel coerced into admission continue to feel coerced during hospital treatment, (c) to identify factors associated with feelings of coercion during treatment, and (d) to explore what experiences—in the view of the patients—lead to feelings of coercion both at admission and during treatment.	UK	Inpatient	Voluntarily admitted patients in nine acute wards in two hospitals in East London	270	Mixed methods	Sociodemographic characteristics, clinical characteristics, global assessment of functioning, and satisfaction with care	McArthur Perceived Coercion Scale (MPCS), Coercion Ladder (CL)
**Tschopp et al. (2011)** [90]	The purpose of the present study was to evaluate the degree of coercion perceived by mental health consumers in an (Assertive Community Treatment) ACT program, the extent to which coercive strategies are perceived to be implemented, and how perceived coercion might relate to the variables of quality of life, working alliance, and sense of empowerment.	USA	Outpatient	Adults diagnosed with predominantly schizophrenia and schizoaffective disorders in an ACT program in the Midwest of the USA	65	Mixed methods	Sociodemographic characteristics and legal status	N/A
**Castille et al. (2011)** [91]	In the present chapter we begin with a comparison between the two previously mentioned approaches to measuring coercion. We ask: does a person with a court order perceive him/herself as being more coerced than a person in outpatient treatment without a court order?	USA	Outpatient	Men and women between the ages of 18 and 65 years with a history of serious mental illness from various outpatient clinics in two boroughs of New York City	184	Mixed methods	Sociodemographic characteristics, clinical characteristics, quality of life, legal status, and previous contact with services	MacArthur Admission Experience Survey (AES)
**Sheehan & Burns (2011)** [15]	The aim of the study was to investigate the association between the therapeutic relationship and perceived coercion in psychiatric admissions.	UK	Inpatient	Patients admitted to psychiatric hospital	164	Quantitative	Therapeutic alliance, sociodemographic characteristics, and legal status	MacArthur Admission Experience Survey (AES)
**Galon & Wineman (2011)** [92]	This study aimed to compare OPC and ACT as forms of coercive interventions to evaluate the influence of each individually and in combination on treatment compliance and client-centered outcomes, including quality of life, symptom distress, empowerment, violence, and victimization.	USA	Outpatient	Individuals with severe and persistent mental health problems	154	Quantitative	Outpatient commitment and assertive community treatment	MacArthur Admission Experience Survey (AES)
**Zuberi et al. (2011)** [93]	This article explores perceptions of coercion and hospitalization among patients admitted to a psychiatric unit in Karachi. We looked for associated patient characteristics.	Pakistan	Inpatient	Patients admitted to psychiatric hospital	87	Quantitative	Legal status and sociodemographic characteristics	MacArthur Admission Experience Survey (AES)
**Newton-Howes & Stanley (2012)** [5]	The aim of this review is therefore to systematically collate those papers that outline the prevalence of perceived coercion to ascertain how common this is and understand the variation in reported rates. An exploration of the factors that may increase or decrease these rates from both a methodological and an epidemiological perspective is also considered.	New Zealand	Mixed	Papers describing adults between 16 and 65 years of age in adult psychiatric care	18	Literature review and meta-regression	Legal status, geographical study location, and instruments used to measure coercion	N/A
**Theoridou et al. (2012)** [14]	The aim of the present study was to investigate the relationship between perceived coercion and the therapeutic relationship. We hypothesized that perceived coercion would negatively influence the therapeutic relationship as rated by the patient, and vice versa. Thus, we did not posit a unidirectional but a reciprocal effect. We further hypothesized perceived coercion to be influenced by legal status, both at admission and in previous hospitalizations.	Switzerland	Inpatient	Patients admitted to psychiatric hospitals	116	Quantitative	Sociodemographic characteristics, clinical characteristics, global assessment of functioning, and legal status	MacArthur Admission Experience Survey (AES)
**Del Vecchio et al. (2012)** [94]	This study, conducted within the EUNOMIA project on the evaluation of coercive measures in psychiatry in twelve European countries, intended to assess (1) the clinical and socio-demographic characteristics most frequently associated with higher levels of perceived coercion at admission (2) the relationship between psychiatric symptoms and levels of perceived coercion.	Italy	Inpatient	Patients admitted to psychiatric wards in twelve European countries (Bulgaria, Czech Republic, Germany, Greece, Israel, Italy, Lithuania, Poland, Slovakia, Spain, Sweden, and the United Kingdom)	2815	Quantitative	Clinical characteristics, and social functioning	McArthur Perceived Coercion Scale (MPCS)
**Galon et al. (2012)** [95]	This study aimed to examine if assignment to OPC status or ACT differed by race and to elucidate the effect of race on the perceptions of procedural justice and coercion in persons subject to OPC.	USA	Outpatient	Patients placed under outpatient commitment (OPC) orders	140	Quantitative	Sociodemographic characteristics, and procedural justice	MacArthur Admission Experience Survey (AES)
**Fiorillo et al. (2012)** [96]	This study aimed to identify (1) sociodemographic and clinical characteristics associated with perceived coercion at admission and (2) changes in symptoms and global functioning associated with changes in perceived coercion over time.	Italy	Inpatient	Patients who were involuntarily admitted or who felt coerced into hospital treatment despite a legally voluntary admission	3093	Mixed methods	Sociodemographic characteristics, clinical characteristics, global assessment of functioning, legal status, and previous contact with services	McArthur Perceived Coercion Scale (MPCS), Cantril Ladder of Perceived Coercion
**Morcos (2012)** [97]	This paper aimed to examine the relationship between interpersonal style, attitude to medication and potential adherence, and perceived coercion.	UK	Inpatient	Adult male inpatients from general and forensic psychiatry wards treated with antipsychotic medication	52	Quantitative	Interpersonal style and treatment compliance	MacArthur Admission Experience Survey (AES)
**Jaeger et al. (2013)** [98]	This paper aimed to examine the long-term influence of involuntary hospitalization on medication adherence, engagement in outpatient treatment and perceived coercion to treatment participation.	Germany	Inpatient	Hospitalized patients with schizophrenia or schizoaffective disorder	374	Quantitative	Legal status, sociodemographic characteristics, and clinical characteristics	Compliance Self-Rating Instrument (CSRI-K)
**Anestis et al. (2013)** [99]	This study aims to elucidate the characteristics of patients and their admissions that increase the likelihood of perceived coercion during short-term psychiatric hospitalization. It seeks to determine whether interpersonal style—specifically Hostile (H), Dominant (D), and Hostile–Dominant (H–D) styles, which have previously been associated with adverse reactions to hospitalization—along with psychiatric symptoms, gender, and admission status (voluntary or involuntary), predict perceived coercion. Additionally, the study investigates whether the perception of coercion at admission remains stable one year after hospitalization.	Australia	Inpatient	Inpatients admitted to the two acute units at the Alfred Hospital Inpatient Psychiatry Department, in Melbourne, Australia, between 1 March 2009 and 10 August 2009	125	Mixed methods	Legal status, sociodemographic characteristics, and clinical characteristics	MacArthur Admission Experience Survey (AES)
**McNiel et al. (2013)** [100]	The study aimed to evaluate the hypothesis that aspects of the treatment relationship, such as the working alliance, psychological reactance, and perceived coercion, could be important in understanding treatment adherence and satisfaction in a group of patients at risk of experiencing leverage.	USA	Outpatient	Outpatients at two community mental health centers	198	Quantitative	Working alliance quality, psychological reactance, leverage, sociodemographic characteristics, and clinical characteristics	MacArthur Perceived Coercion Scale (MPCS), adapted for outpatient treatment
**Seo et al. (2013)** [101]	This study analyzed whether coercive intervention observed in Korea’s field of mental health could be justified by the basic assumptions of paternalists: the assumptions of incompetence, dangerousness, and impairment.	South Korea	Inpatient	Patients who had been hospitalized following diagnoses of schizophrenia or mood disorders stayed in the hospital for four weeks	248	Quantitative	Sociodemographic characteristics, clinical characteristics, and global assessment of functioning	MacArthur Admission Experience Survey (AES)
**Terkelsen & Larsen (2013** [102]**)**	This study aimed to explore how patients and staff act in the context of involuntary commitment, how interactions are described, and how they might be interpreted.	Norway	Inpatient	People with mental health and substance abuse problems	38	Qualitative	Sociodemographic characteristics, clinical characteristics, and characteristics of the therapeutic environment	N/A
**Newton-Howes et al. (2014)** [103]	This study aimed to investigate the experience of community treatment orders (CTOs) among Maori and non-Maori patients, comparing their views within mainstream and Maori mental health services.	New Zealand	Outpatient	Patients with experience of CTOs	79	Quantitative	Legal status, sociodemographic characteristics, and type of mental health service	MacArthur Perceived Coercion Scale (MPCS)
**Larsen & Terkelsen (2014)** [104]	This brief literature review gives a few examples of differences in staff and patient attitudes related to coercion, shows how forced treatment might weaken the alliance between staff and patients, and suggests dialogue and reciprocity as practices to reduce coercion.	Norway	Inpatient	Patients from an inpatient psychiatric unit in Norway	12	Qualitative	Legal status and sociodemographic characteristics	N/A
**O’Donoghue et al. (2014)** [105]	In this study we aimed to quantify the proportion of voluntarily admitted service users with levels of perceived coercion equivalent to that of involuntarily admitted service users. Secondly, we aimed to identify demographic and clinical characteristics of voluntarily admitted service users who experienced high levels of perceived coercion.	Ireland	Inpatient	Individuals admitted voluntarily and involuntarily to three psychiatric hospitals	161	Quantitative	Legal status, clinical characteristics, negative pressures, and procedural justice	MacArthur Perceived Coercion Scale (MPCS)
**Riley et al. (2014)** [106]	The objective of this qualitative study was to explore (1) patients’ experiences with OC (outpatient commitment) and (2) how routines in care and health services affect patients’ everyday living.	Norway	Outpatient	Patients under an OC order who had been at least under the order for 3 months and lived in the catchment area	11	Qualitative	Accommodation (staffing, supervision, etc.), frequency of monitoring by clinicians, initial contact with mental health services, and clinical characteristics	N/A
**Munetz et al. (2014)** [107]	The study aimed to examine levels of perceived coercion, procedural justice, and the impact of mental health court (MHC) and assisted outpatient treatment (AOT) programs among participants in a community treatment system.	USA	Outpatient	Individuals who had graduated from a mental health court program and former AOT participants who were no longer under court supervision	52	Quantitative	Interactions with judges and case managers and procedural justice	Modified MacArthur Perceived Coercion Scale (MPCS)
**Prebble et al. (2015)** [21]	This review aimed to identify literature pertaining to the experiences of people admitted voluntarily to acute adult mental health facilities.	New Zealand	Inpatient	Publications focused on the experiences of voluntary service users in acute adult psychiatric facilities	46	Review of the literature	Legal status, perception of coercion, procedural justice, knowledge of rights, and informed consent	N/A
**Donskoy (2015)** [108]	The purpose of this paper is to present a focused viewpoint of coercion in psychiatry from the perspective of a survivor and activist.	UK	Inpatient	Psychiatric inpatients	N/A	Viewpoint of a psychiatric survivor and human rights activist	Lack of capacity, consent, paternalism, complicit psychiatry, and application of human rights standards	N/A
**Canvin (2016)** [109]	This chapter presents a synthesis of major research themes and findings on patients’ subjective experiences and perceptions of coercion in community psychiatry.	Unspecified	Outpatient	Patients in community psychiatric settings	N/A	Book chapter: synthesis of the literature	Interventions (medication, appointments); obligations (institutional rules, treatment plans, providers’ expectations); threats; and safety	N/A
**Norvoll & Pedersen (2016)** [110]	This study aimed to explore the views of people with mental health problems on the concept of coercion and to argue for a broader socio-ethical perspective on coercion in mental health care.	Norway	Mixed	Adults with various mental health problems and experiences with coercion	24	Qualitative	Formal and informal coercion across health and welfare services, power relations, deprivation of freedom, and social and existential impacts of coercion	N/A
**Fugger et al. (2016)** [111]	The objective of the present longitudinal investigation was to analyze the burden caused by physical restraint in psychiatric wards. Three specific research questions were addressed: (1) Does the patients’ subjective perception of physical restraint change the longer the time span from the last fixation? (2) Is there a difference between the patients’ and the investigators’ evaluation of physical restraint? (3) Is there a connection between physical restraint and the presence of consecutive posttraumatic stress disorder?	Austria	Inpatient	Patients who were involuntarily admitted and physically restrained at a psychiatric intensive care unit in the general hospital of Vienna (AKH)	47	Quantitative	Influence on the treatment process, self-evaluation of physical restraint, symptoms of post-traumatic stress disorder, clinical characteristics, and clinical global impression	MacArthur Admission Experience Survey (AES)
**Francombe Pridham et al. (2016)** [22]	This review of literature aimed to examine the relationship between community treatment orders (CTOs) and patients’ perceptions of coercion, with the objective of understanding factors that might influence the experience of being placed under a CTO.	Canada	Outpatient	Publications focused on the experiences of patients who were or had been subject to a community treatment order (CTO)	23	Review of the literature	Clinical history and characteristics, procedural justice, the legal and health services context of CTOs, presence of additional forms of leverage, and communication with service providers	N/A
**Raveesh et al. (2016)** [112]	This study aimed to assess perceived coercion in persons with mental disorders admitted involuntarily and correlate it with sociodemographic factors and illness variables.	India	Inpatient	Patients admitted involuntarily to a psychiatric hospital	301	Quantitative	Sociodemographic characteristics, clinical characteristics, and coercive measures	MacArthur Admission Experience Survey (AES)
**Zlodre et al. (2016)** [113]	The current study examined competence and coercion in a cohort of individuals with severe personality disorder who were detained in high-security hospital and prison settings.	UK	Inpatient	Individuals with severe personality disorder who were detained in high-security hospital and prison settings	174	Quantitative	Clinical characteristics, competence to consent to treatment, and coercion	MacArthur Admission Experience Survey (AES)
**Abt (2016)** [114]	The research question guiding this dissertation is to explore patients’ reactions to coercion. How does it influence their behavior in the relationship they have with healthcare providers? Specifically, the aim is to understand how the patient processes their experience of coercion and how they manage it.	Switzerland	Inpatient	Involuntarily admitted patients	11	Qualitative	Patients’ experiences of involuntary hospitalization and therapeutic alliance	N/A
**Opsal et al. (2016)** [115]	The present study aimed to investigate the role that perceived coercion played among patients with SUD that entered treatment. We also aimed to clarify whether patients that were admitted involuntarily perceived coercion differently from those that were admitted voluntarily and to identify factors that could predict perceived coercion.	Norway	Inpatient	Voluntarily and involuntarily admitted patients	192	Mixed methods	Sociodemographic characteristics, clinical characteristics, and legal status	Perceived Coercion Questionnaire (PCQ)
**Gowda et al. (2016)** [116]	The main questions of this study were (1) which coercive measures were taken? (2) What was the perceived coercion at admission and at discharge? (3) Which patient and contextual characteristics were related to perceived coercion at admission and discharge?	India	Inpatient	Patients admitted to psychiatric hospitals	75	Quantitative	Sociodemographic characteristics, clinical characteristics, and clinical global impression	MacArthur Perceived Coercion Scale (MPCS), Coercion Ladder (CL)
**Gowda et al. (2017)** [117]	We aimed to study coercion experiences among patients with schizophrenia who were admitted involuntarily. Additionally, we also assessed if demographic factors, clinical factors, and the use of coercive measures had any influence on how patients perceived the necessity of their own involuntary admission.	India	Inpatient	Patients with schizophrenia admitted under special circumstances of the Mental Health Act	76	Quantitative	Sociodemographic characteristics and clinical characteristics	MacArthur Admission Experience Survey (AES)
**O’Donoghue et al. (2017)** [10]	The ‘Service Users’ Perspective of their Admission’ study examined voluntarily and involuntarily admitted service users’ perception of coercion during the admission process and whether this was associated with factors such as the therapeutic alliance, satisfaction with services, functioning, and quality of life. This report aims to collate the findings of the study.	Australia	Inpatient	Involuntarily admitted patient to three psychiatric inpatient units in Dublin and Wicklow	161	Quantitative	Sociodemographic characteristics and clinical characteristics	MacArthur Admission Experience Survey (AES)
**Larkin & Hutton (2017)** [23]	This systematic review and meta-analysis aimed to determine the direction, magnitude, and reliability of the relationship between capacity in psychosis and a range of clinical, demographic, and treatment-related factors, thus providing a thorough synthesis of current knowledge.	UK	Unspecified	Publications focused on adults diagnosed with a non-affective psychotic disorder	23	Systematic review, meta-analytical and narrative synthesis	Sociodemographic characteristics, clinical characteristics, and perceived coercion	N/A
**Anonymous (2017)** [118]	This article presents a patient’s experience with psychiatric services.	Lebanon	Mixed	Psychiatric service user	N/A	First-person account	Legal status and relationship with staff	N/A
**Kisely et al. (2017)** [119]	This systematic review aims to examine the effectiveness of compulsory community treatment (CCT) for people with severe mental illness (SMI).	Australia	Outpatient	Trials of adults with SMIs who were managed in a community setting	3	Systematic review	Comparison I: compulsory community treatment versus entirely voluntary care; comparison II: community treatment orders versus supervised discharge; comparison III: community treatment orders versus standard care	N/A
**Ramachandra et al. (2017)** [120]	The present study was aimed at investigating the perceived coercion of psychiatric patients during admission into a psychiatric hospital, keeping the current Mental Health Act 1987 and MHC Bill, 2013, in perspective.	India	Inpatient	Voluntarily and involuntarily admitted patients	205	Quantitative	Sociodemographic characteristics, clinical characteristics, legal status, and history of past hospitalization	MacArthur Admission Experience Survey (AES)
**Bradbury et al. (2017)** [121]	The aim of the current study was to provide an understanding of the lived experience of involuntary transport (under the MHA) from the perspectives of consumers, carers, mental health nurses, police officers, and ambulance paramedics.	Australia	Inpatient	People with the lived experience of involuntary transport under the MHA: consumers, carers, mental health nurses, police officers, and ambulance paramedics	16	Qualitative	Perspectives of consumers, carers, mental health nurses, police officers, and ambulance paramedics	N/A
**Tomlin et al. (2018)** [122]	This systematic review examines both empirical and policy literature with the aim of conceptualizing the restrictiveness of forensic care as described by residents, staff, and academic commentators.	UK	Inpatient	Papers were included if they were conducted in secure forensic facilities and involved mentally disordered offenders aged over 18 with any clinical diagnosis	50	Systematic review and concept analysis	Phenomenon of restrictiveness as experienced through relationships, institutional characteristics, and systemic factors	N/A
**Horvath et al. (2018)** [123]	The present study examines forensic psychiatric inpatients’ perception of coercion regarding the prescribed antipsychotic medication and factors associated with the perception of coercion.	Germany	Inpatient	Patients with schizophrenia, schizotypal, and delusional disorders in two forensic psychiatric institutions in Southern Germany	56	Quantitative	Sociodemographic characteristics and clinical characteristics	MacArthur Admission Experience Survey (AES), Coercion Ladder (CL), Coercion Experience Scale (CES)
**Nakhost et al. (2018)** [24]	This study aimed to assess the perception of coercion among service users treated under a community treatment order (CTO) compared to a matched comparison group of voluntary psychiatric outpatients and examined the potential predictors of perceived coercion.	Canada	Outpatient	Service users treated under a CTO; voluntary psychiatric outpatients	138	Quantitative	Sociodemographic characteristics, clinical characteristics, legal status, procedural justice, and perceived coercion	N/A
**Gowda et al. (2018)** [124]	This article aimed to study the prevalence of restraint in an Indian psychiatric inpatient unit and to examine the level of perceived coercion correlating to various forms of restraint.	India	Inpatient	Psychiatric inpatients	200	Qualitative	Sociodemographic characteristics, clinical characteristics, legal status, history of past hospitalization, and coercive measures	N/A
**Allison & Flemming (2019)** [125]	The aim of this review was to explore mental health patients’ treatment-related experiences of softer coercion and its effect on their interactions with practitioners through a synthesis of qualitative research. There are two main objectives (1) identify patients’ experiences of soft/subtle coercion during admission to, or in, treatment in mental health services and (2) explore the perceived effect of this coercion on patient–practitioner interactions.	UK	Inpatient	Papers describing experiences of patients in mental health services	11	Qualitative thematic synthesis	Sense of self, therapeutic alliance and patients’ perception about their transition through treatment	N/A
**Sampogna et al. (2019)** [13]	The aim of the study was to (1) identify the sociodemographic and clinical characteristics associated with high levels of perceived coercion at admission in psychiatric wards; (2) assess the relationship between the levels of perceived coercion at admission and the levels of satisfaction with received care after three months of hospitalization in a sample of Italian patients with severe mental disorders.	Italy	Inpatient	Patients admitted to psychiatric hospitals	294	Quantitative	Legal status, sociodemographic characteristics, and satisfaction with care	MacArthur Perceived Coercion Scale (MPCS), Cantril Ladder of Perceived Coercion Scale
**Lamothe et al. (2019)** [126]	The primary objective was to measure the relationship between coercive stress experienced by patients hospitalized in the psychiatric intensive care unit and their level of insight in order to identify potential psychotherapeutic approaches to improve their experience of care and, consequently, enhance clinical practice. The secondary objective was to highlight a potential link between specific coercive measures (such as seclusion) and coercive stress.	France	Inpatient	Patients who had been hospitalized in the psychiatric intensive care unit of the Caen University Hospital	40	Quantitative	Sociodemographic characteristics and clinical characteristics	Coercion Experience Scale (CES)
**Guzmán-Parra et al. (2019)** [127]	The objective of this study was to analyze the patients’ perceived coercion, symptoms of post-traumatic stress, and subjective satisfaction with the hospitalization treatment associated with the use of different coercive measures during psychiatric hospital stays, particularly the use of involuntary medication, mechanical restraint, or a combination of these measures.	Spain	Inpatient	Patients who had been subject to coercive measures during their psychiatric hospitalization in the Mental Health Hospitalization Units of the University Regional Hospital of Malaga and the General Hospital of Jerez de la Frontera	111	Quantitative	Sociodemographic characteristics, clinical characteristics, coercive measures, satisfaction with treatment, post-traumatic stress, and perceived stress	Coercion Experience Scale (CES), Coercion Ladder (CL)
**Akther et al. (2019)** [128]	The aim of this systematic review was to synthesize qualitative evidence of patients’ experiences of being formally assessed for admission and/or the subsequent experience of being detained under mental health legislation. This included any legal processes that take place during the assessment process and during detention, such as mental health tribunals.	UK	Inpatient	Papers describing patients’ experiences of assessment or detention under mental health legislation	56	Systematic review and qualitative meta-synthesis	Information and involvement in care, therapeutic environment, relationships with staff, and impact of detention on self-worth and emotional well-being	N/A
**Golay et al. (2019)** [129]	The first objective of this study was to disentangle the respective contributions of legal admission status and perceived admission status in predicting perceived coercion. The second objective was to examine the extent to which the perceived usefulness of hospitalization influenced perceived coercion.	Switzerland	Inpatient	Patients hospitalized in the Department of Psychiatry at Lausanne University Hospital	152	Mixed methods	Perceived legal status, perceived need for hospitalization, and subjective improvement	MacArthur Admission Experience Survey (AES), Coercion Experience Scale (CES)
**Gerle et al. (2019)** [130]	The aim of this study was to investigate how 6 patients, who had received care for self-injurious behavior, perceived coercion and how they think coercion could be avoided.	Sweden	Inpatient	Patients who had engaged in self-injury and had been subjected to coercive measures during treatment	6	Qualitative	Threats, coercive measures, control, and involvement in care	N/A
**Mandarelli et al. (2019)** [131]	The aim of the present study was to translate into Italian and validate the Admission Experience Survey (I-AES) and to explore its psychometric properties, including factorial structure. A second objective of the study was to investigate differences in perceived coercion in different diagnostic groups of psychiatric patients as well as in voluntarily and involuntarily hospitalized patients.	Italy	Inpatient	Voluntarily and involuntarily admitted patients	156	Quantitative	Sociodemographic characteristics and clinical characteristics	MacArthur Admission Experience Survey (Italian version)
**Clément et al. (2020)** [132]	The objective of this study is to identify differences in perceptions between nurses and patients regarding their ideal relationship in the context of a first psychotic episode.	Canada	Inpatient	Individuals who experienced involuntary hospitalization in the context of a first psychotic episode; nurses	10	Qualitative	Therapeutic alliance	N/A
**Jessell (2020)** [133]	This dissertation aimed to understand the relationship between recovery orientation and the role of psychiatric medication in service planning.	USA	Mixed	Users of mental health services	731	Mixed methods	Sociodemographic characteristics, clinical characteristics, recovery orientation of the plan, and role of psychiatric medication	N/A
**Scholes et al. (2021)** [134]	The aim of this systematic review was to synthesize women service users’ experiences of inpatient mental health services and staff experiences of providing care to women within inpatient mental health services, to appraise the methodological quality of research in this area, and to provide recommendations for clinical practice and future research.	UK	Inpatient	Papers describing the experiences of women service users in inpatient mental health services or staff experiences of providing care to women in these settings	18	Systematic review of qualitative evidence	Feeling of safety, perceptions of factors contributing to iatrogenic harm and ineffective inpatient care, and therapeutic environment	N/A
**Schoppmann et al. (2021)** [135]	This explorative study investigates baseline expectations and views of patients in forensic wards in German-speaking Switzerland in the context of a recovery-oriented intervention.	Switzerland	Inpatient	Forensic inpatients	37	Qualitative	Sociodemographic characteristics, clinical characteristics, length of stay, and reasons for being sentenced	N/A
**Lee & Seo (2021)** [136]	This study analyzed the effect of the perceived coercion of persons with mental illness who use community mental health services in Korea on their therapeutic satisfaction and their life satisfaction mediated by the therapeutic relationship.	South Korea	Outpatient	Outpatients with mental disorders aged over 20 years	185	Quantitative	Sociodemographic characteristics, clinical characteristics, therapeutic alliance, life satisfaction, and satisfaction with care	MacArthur Admission Experience Survey (AES)
**O’Callaghan et al. (2021)** [137]	This study aims to determine the relationships, if any, between perceived coercion on admission and subsequent formal coercive practices during the admission among psychiatry inpatients in Ireland, and any relationships between perceived coercion on admission and other variables such as age, gender, and diagnosis.	Ireland	Inpatient	Voluntary and involuntary psychiatric inpatients aged 18 years or older who were admitted to the acute psychiatric admission units at Tallaght University Hospital and Connolly Hospital in Dublin	107	Quantitative	Sociodemographic characteristics, clinical characteristics, legal status, length of stay, level of functioning, and coercive measures	MacArthur Admission Experience Survey (AES)
**Fiore et al. (2021)** [138]	This study reviews qualitative studies about long-acting injectable antipsychotic drugs with the aim to gain a better understanding of how LAI therapy is perceived by patients, to analyze the perceived pros and cons of treatment with LAIs in comparison to oral medication, and to investigate factors in favor of each particular method of administration based on the experience and opinions of patients.	Italy	Inpatient	This study includes qualitative research exploring patients’ subjective experiences and attitudes toward long-acting injectables (LAIs)	220	Systematic review of qualitative studies	Information received, therapeutic alliance, attitude toward LAIs, coercion, stigma, and recovery	N/A
**Hirsch et al. (2021)** [139]	The present study investigates perceived coercion in psychiatric inpatients under prescribed antipsychotic medication without a court order. The objective of this study was to investigate whether and to what extent involuntary and voluntary inpatients feel coerced to take their medication and which factors affect perceived coercion.	Germany	Inpatient	Voluntarily and involuntarily admitted patients	91	Quantitative	Sociodemographic characteristics, clinical characteristics, and legal status	MacArthur Admission Experience Survey (AES)
**Mandarelli & Parmigiani (2021)** [140]	Tools for health professionals to improve their communication skills; context: in North America principles of informed consent, patient’s autonomy, and case law have established ethical and legal obligations to provide patients with as much information as they desire about their illness and its treatment [7].	Italy	Unspecified	Psychiatric patients at risk of impaired capacity to consent to treatment	N/A	Book chapter	Legal status and clinical characteristics	N/A
**Tully et al. (2022)** [141]	The study aimed to explore women’s experiences of routine restrictive practices in mental health inpatient settings.	UK	Inpatient	Women who were inpatients on psychiatric wards	22	Qualitative	Impact of restrictions on relationships, influence on the treatment process, and restrictions providing safety and support	N/A
**Bendall et al. (2022)** [142]	The study aimed to explore experiences of restrictive practices from the perspectives of acute care inpatients and staff.	UK	Inpatient	Acute psychiatric inpatients and staff	17	Qualitative	Exploring participants’ experiences of situations considered to involve restrictive practices	N/A
**Smyth et al. (2022)** [143]	This study aimed to examine and compare retrospective qualitative perceptions of service users in relation to their involuntary admission with their levels of clinical insight, using a mixed-methods approach.	Ireland	Inpatient	Involuntarily admitted inpatients	42	Mixed methods	Sociodemographic characteristics, clinical characteristics, and level of insight	N/A
**Plunkett et al. (2022)** [144]	This research aimed to provide quantitative data about self-rated dignity among involuntary and voluntary psychiatry inpatients and to explore relationships between perceived dignity and legal status, coercion, level of insight, diagnosis, and therapeutic alliance.	Ireland	Inpatient	Psychiatric inpatients aged 18 years or older	107	Quantitative	Legal status, level of insight, negative symptoms, procedural justice, and affective reactions to hospitalization	MacArthur Admission Experience Survey (AES)
**Jina-Pettersen (2022)** [145]	This study aimed to further understand experiences of inpatient psychiatric trauma using an anonymous platform to obtain forthright data.	Netherlands	Inpatient	Psychiatric inpatient	262	Qualitative	Patients’ lived experiences and subjective perspectives during psychiatric hospitalization	N/A
**Martinez et al. (2022)** [146]	The present study aimed to assess the impact of past experience of coercion on the perception of coercion and satisfaction with subsequent voluntary hospitalizations.	Switzerland	Inpatient	Psychiatric patients from six hospitals in the French-speaking region of Switzerland	140	Quantitative	Sociodemographic characteristics, satisfaction regarding hospitalization, health and social functioning, and self-reported health	The MacArthur Admission Experience Survey (AES), Coercion Experience Scale (CES)
**Hotzy et al. (2023)** [147]	The aim of this study was to analyze whether involuntarily admitted patients in different psychiatric hospitals in Switzerland feel well informed about IA and whether the level of perceived information is associated with perceived coercion.	Switzerland	Inpatient	Involuntarily admitted patients	224	Quantitative	Sociodemographic characteristics and experience with involuntary admission	MacArthur Admission Experience Survey (AES)
**Silva et al. (2023)** [148]	This review aimed to provide an aggregative synthesis of the qualitative evidence on patients’ experienced coercion during voluntary and involuntary psychiatric hospitalization.	Switzerland	Inpatient	Voluntarily and involuntarily admitted patients	26	Review and meta-aggregation of qualitative studies	Patients’ experience of the hospitalization and the associated feeling of coercion, involvement in the decision-making process, and relationships with the staff	N/A
**Carimbocas (2023)** [149]	The present mixed-method dissertation study examined the potential association between different levels (high or low) of the MacArthur Admission Experience Scale (MAES) variables (perceived coercion, negative pressures, and procedural justice) and recovery outcomes, which were measured using the Illness Management and Recovery Scale (IMRS).	USA	Inpatient	Adults (18 years and older) who had experienced at least one psychiatric hospitalization in their lifetime	31	Mixed methods	Sociodemographic characteristics, clinical characteristics, admission experience, and inpatient experience	MacArthur Admission Experience Scale (AES)
**Wullschleger et al. (2023)** [150]	The present study aims to analyze the relationship between patients’ appraisal of the justification of coercive measures and their level of perceived coercion.	Germany	Inpatient	Psychiatric inpatients	97	Quantitative	Sociodemographic characteristics, clinical characteristics, and coercive measures	Coercion Experience Scale (CES)
**O’Callaghan et al. (2023)** [151]	This paper explores factors linking gender with increased perceived coercion, perceived negative pressures, and procedural injustice during psychiatric admission.	Ireland	Inpatient	Adult psychiatric inpatients admitted to acute psychiatric admission units at two general hospitals in Dublin, Ireland	107	Quantitative	Sociodemographic characteristics, legal status, and coercive measures	MacArthur Admission Experience Survey (AES)
**Pelosse (2023)** [152]	The aim of this paper was to gain a deeper understanding of the experiences of coercion and support in exercising rights as lived by individuals with mental health issues who are hospitalized or treated involuntarily in psychiatric care.	Canada	Inpatient	Adult individuals with a history of involuntary psychiatric hospitalization or treatment	11	Qualitative	Sociodemographic characteristics, coercion, and support in the exercise of rights	N/A
**Silva et al. (2023)** [153]	This study aimed to explore voluntary and involuntary patients’ experience of coercion during psychiatric hospitalization and to identify which factors, from their perspective, most affected it.	Switzerland	Inpatient	Inpatients aged between 18 and 65 years who had been hospitalized for more than seven days but less than 15 days	12	Qualitative	Relationship with staff, therapeutic environment, and institutional rules	N/A
**Shozi et al. (2023)** [154]	The aim of this study was to describe the patient’s experiences of involuntary admission at two psychiatric hospitals in KwaZulu-Natal.	South Africa	Inpatient	Involuntary patients aged 18 years and older	131	Quantitative	Sociodemographic characteristics and clinical characteristics	MacArthur Perceived Coercion Scale (MPCS)
**Paun et al. (2024)** [155]	The present study aims to evaluate the psychometric properties of a Romanian language version of the AES (R-AES) and to identify predictors for higher perceived coercion.	Romania	Inpatient	Patients admitted to the ‘Professor Doctor Alexandru Obregia’ Psychiatric Hospital in Bucharest	112	Quantitative	Sociodemographic characteristics and clinical characteristics	McArthur Admission Experience Survey (Romanian version)
**Morandi et al. (2024)** [156]	This study examined how inpatients’ involvement in the decision-making process, the respect of their decision-making preference, and their feeling of having been treated fairly mediate the relationship between involuntary hospitalization and perceived coercion both at admission and during hospital stay.	Switzerland	Inpatient	Voluntary and involuntary hospitalized patients across six psychiatric hospitals in the French-speaking part of Switzerland	230	Quantitative	Legal status, involvement in decision-making, respect for patients’ decision-making preferences, and patients’ perceived fairness of treatment pressures	MacArthur Admission Experience Survey (AES) and Coercion Experience Scale (CES)
**Indregard et al. (2024)** [157]	In this study, we aimed to compare the occurrence of coercion in open-door policy wards and locked treatment-as-usual wards.	Norway	Inpatient	Psychiatric inpatients at Lovisenberg Diaconal Hospital	556	Quantitative	Open-door policy	Experienced coercion scale (ECS)
**Silva et al. (2024)** [158]	This study aimed to explore the interplay between these factors and to provide new insights into how they lead to experienced coercion.	Switzerland	Inpatient	Voluntarily and involuntarily admitted patients	225	Quantitative	Formal and informal coercion, perceived fairness and effectiveness, implication in decision-making, and satisfaction with hospitalization	MacArthur Admission Experience Survey (AES)
**Fossum et al. (2024)** [159]	This study aimed to investigate the association between patient-reported mental health care quality, perceived coercion, and various demographic, clinical, and ward-related factors.	Norway	Inpatient	Psychiatric inpatients	169	Quantitative	Quality of care, sociodemographic characteristics, clinical characteristics, and therapeutic environment	Experienced coercion scale (ECS)
**Aluh et al. (2025)** [160]	This study aimed to investigate the subjective experience of coercion among patients on admission in Portuguese psychiatric departments by assessing their perceived coercion, procedural justice, and negative pressures during admission. The study also investigated whether this subjective experience of coercion changed with time during admission, and the predictors of this change.	Portugal	Inpatient	Adults admitted to five public psychiatric inpatient departments in rural and urban regions of Portugal	208	Quantitative	Procedural justice, time, legal status, satisfaction with care, gender, number of previous admissions, and immigrant status	McArthur Admission Experience Survey (AES), Coercion Ladder (CL)
**Aragones-Calleja & Sánchez-Martínez (2025)** [161]	This study aimed to describe and measure users’ experience of coercion and explore their perception of the treatment received in an inpatient medium-stay psychiatric rehabilitation unit (IMSPRU).	Spain	Inpatient	Patients hospitalized in a medium-stay psychiatric rehabilitation unit	75	Mixed methods	Relationship with staff and formal and informal coercion	Coercion Experience Scale (CES)
**Nakhost et al. (2025)** [162]	This research seeks to address knowledge gaps by studying the prevalence of current experiences of various clinical leverages among general and community mental health outpatients and assessing the relationship between such current clinical leverages and subjective perception of coercion.	Canada	Outpatient	General and community mental health outpatients	137	Quantitative	Sociodemographic characteristics, clinical leverages, pressures, perceived coercion, and clinical characteristics	N/A
**Hudson & Beames (2025)** [163]	This lived experience narrative recounts the first author’s week-long stay in a psychiatric ED, providing insight into the experiences and challenges of inpatient psychiatric care.	Canada	Inpatient	Psychiatric service user	N/A	Lived experience narrative	Experiences and challenges of inpatient psychiatric care	N/A

Published between 1993 and 2025, the included literature is primarily in English, with the exception of four in French, and originates from 25 countries: the United States (34), the United Kingdom (21), Switzerland (11), Norway (10), New Zealand (9), Canada (7), Ireland (6), Italy (6), Sweden (6), Australia (5), India (5), Germany (4), South Korea (3), Denmark (2), Spain (2), South Africa (1), Austria (1), Brazil (1), China (1), France (1), Lebanon (1), Pakistan (1), the Netherlands (1), Portugal (1), and Romania (1).

Two are master’s dissertations, and four are doctoral theses. Five are abstracts of presentations delivered at international conferences. Two are book chapters [109,140], one is a lived experience narrative [163], another is a first-person account [118], and one is a focused viewpoint on coercion in psychiatry [108]. The remaining 127 are research articles, including 16 reviews. Regarding methodologies, 25 studies use a qualitative design, 24 employ a mixed-methods approach, and 76 follow a quantitative design (including prospective cohort studies, randomized controlled trials, quasi-experimental studies, prospective pilot studies, cross-sectional observational or naturalistic studies, and correlational studies). The study settings are primarily inpatient (100), but also outpatient (31) or mixed (9).

Perceived coercion was assessed using validated scales in 89 of the 143 writings, with the exception of qualitative studies, systematic and literature reviews, book chapters, and 8 quantitative studies. The MacArthur Admission Experience Survey (including the MacArthur Perceived Coercion Scale subscale) was used in 74 writings. Among the 89 writings, 14 used the Coercion Ladder or another Visual Analogue Scale of perceived coercion. Eight studies used the Coercion Experience Scale, and two used the Experienced Coercion Scale. Other studies used the Compliance Self-Rating Instrument (CSRI-K), the Perceived Coercion Questionnaire, the Uppsala Questionnaire on Coercive Measures, and the Survey of Treatment Entry Pressures

### 3.2. Themes

**Figure 2:**
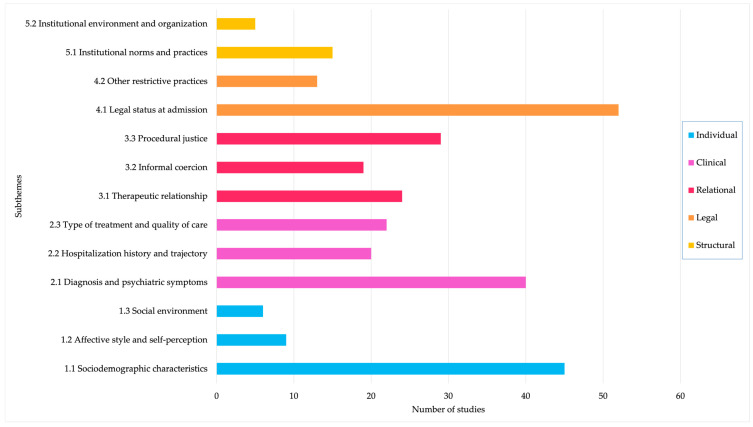
Number of studies addressing the different factors identified in the review.

The presented themes illustrate the various factors studied in the literature on perceived coercion in psychiatry. Five themes emerged from the analysis of the literature and were grouped by the factors they represented: individual, clinical, relational, legal, and structural factors (Figure 2).

#### 3.2.1. Individual

This theme examines how individual characteristics influence the perception of coercion in psychiatric care. These factors include sociodemographic attributes such as age, race, gender, education, income, and employment, as well as personal experiences, emotional states, self-perception, and the role of social networks. While some of these characteristics show associations with perceived coercion, the findings across studies are often inconsistent or context-dependent, highlighting the complexity of interpreting individual-level influences outside of their broader relational and structural contexts.

##### Sociodemographic Characteristics

While some sociodemographic characteristics appear to influence perceived coercion, findings across studies were often inconsistent, with many reporting no significant associations.

Most studies found no significant association between age and perceived coercion [14,15,36,38,41,44,54,65,67,82,85,89,116,130,139,150,155]. However, older age was associated with higher perceived coercion in two studies [64,93], with one specifying that participants over 30 were twice as likely to have high perceived coercion scores [93]. Conversely, younger age was associated with perceived coercion at admission among female patients in one study [151] and among all patients in another [155].

Findings on race or ethnicity (Throughout this article, terms such as race, ethnicity, and other identity-related categories are used as they appeared in the original studies included in the review. These terms reflect the language and classifications employed by the respective authors and do not necessarily reflect the preferred or current terminology.) were mixed; White [42,64,77,149] and Latino [77] patients reported higher levels of perceived coercion than African Americans [41,42,47,77]. One study specifically found that white, single women with higher education were more likely to perceive greater coercion [36]. However, several studies found no significant association between race and perceived coercion [15,65,78,89,103,130,154]. Moreover, no association was found between migration background and perceived coercion [139,160].

Similarly, level of education showed no consistent pattern. Most studies found no significant association between level of education and perceived coercion [14,15,36,41,65,117,130,139,154]. University graduates had significantly higher perceptions of coercion in three studies [45,92,95]. However, one study found that people with lower education levels experienced a greater increase in perceived coercion over time [98].

Marital status findings were inconsistent; having a partner was associated with lower perceived coercion in one study [65], but being married or cohabiting was linked to higher perceived coercion in another [74]. Eight studies found no significant association [15,82,85,117,123,130,139,154].

Sex and gender findings were also inconclusive. Most studies found no significant association [15,38,41,42,44,54,65,67,82,85,117,123,130,139,154,160], though a substantial number reported greater perceived coercion among women [35,36,45,71,89,94,96,99,112,137]. In a systematic review on women’s experiences of inpatient mental health services, it was reported that women often felt their histories of abuse were not considered and that they were coerced into compliance within a fearful environment [134]. Research on sexual and gender diversity was limited, with only one doctoral thesis from the United States finding that LGBTQQIA+ participants experienced higher perceived coercion than cisgender heterosexual participants [149].

Religion was explored in two studies, with one Indian study finding that being Muslim or Christian was associated with increased perceived coercion scores [112], while the other, from New Zealand, found no association with the religion of participants [65].

Findings on socioeconomic status and employment varied. Higher perceived coercion was associated with a high socioeconomic status in one study [116], but with low income [112] or low socioeconomic status [117] in two others. Being employed was associated with lower perceived coercion in three studies [116,117,137]. Several studies found no correlation between perceived coercion and socioeconomic status [41,65] or employment [65,89,117,154]. Living situation was not significantly associated with perceived coercion in any of the seven studies examining this factor [15,36,65,89,117,123,139].

##### Affective Style and Self-Perception

Two studies, one of which included only “male gender” participants [97], found that patients with an interpersonal style characterized as hostile and dominant were more likely to perceive coercion [97,99], while another study found no significant association between interpersonal style and perceived coercion [87]. Emotional responses also played a role in perceptions of coercion. In outpatient care, individuals who experienced sadness, anger, confusion, or fear reported higher levels of perceived coercion, whereas those who felt happiness or relief reported lower levels [65]. Similar findings were observed in inpatient settings, where happiness and relief were associated with lower perceived coercion in both general and forensic populations, while anger and fear were linked to higher levels [55].

Additionally, higher perceived coercion scores were associated with lower self-dignity ratings by patients [144] and among individuals who did not believe they needed treatment or had a mental illness [65] or who felt stigmatized [91]. One study introduced the concept of “internal coercion”, wherein patients felt that their mental illness itself contributed to a sense of loss of control, making hospitalization seem necessary to regain that control [153]. Similarly, a qualitative study reported that feeling ill contributed to patients’ coercive experiences by reinforcing feelings of powerlessness and making it difficult to resist unwanted treatment [110].

##### Social Environment

Social networks appeared to play a complex role in perceived coercion. Two studies found that having extended family was associated with lower perceived coercion [112,116]. However, larger and closely tied social networks could also exert coercion, pressuring individuals to receive treatment [40] or making decisions on their behalf (132). Similarly, close relatives were found to use threats or pressures to facilitate hospital admission [37], even among voluntary patients [60]. Supervisors and co-workers were also vectors of coercive influence in treatment decisions, with one study documenting that they were sometimes the first to encourage or demand the person seek professional help [40].

#### 3.2.2. Clinical

This theme explores how clinical characteristics, such as psychiatric diagnoses, symptom severity, insight, global functioning, treatment history, and quality of care, relate to perceived coercion. It encompasses not only diagnostic categories but also the trajectories of care and treatment modalities, including inpatient, outpatient, and community-based services.

##### Diagnosis and Psychiatric Symptoms

Perceived coercion was associated with bipolar disorder or mood disorders [40,93,155], schizophrenia spectrum disorders [14,85,155], depressive disorders [112], and substance use [93,112]. One study found that a diagnosis of psychotic disorder reduced the likelihood of reporting coercion by six times [93]. An association was found between women who had attempted suicide and higher perceived coercion [71]. Other studies found no significant association with diagnosis [41,54,65,67,89,136,154,162].

While most studies found no association between symptom severity and perceived coercion [38,44,54,67,79,89,99,115,136,139], others reported a positive association [14,105,116,123,131,155,162]. Positive [94,96,137] and negative [131,150] symptoms, delusions of harm [91], and manic excitement/disorganization symptoms [131] were all associated with higher perceived coercion. In contrast, one study found that anxiety and depressive symptoms were negatively associated with perceived coercion [131]. Meanwhile, another study found no association between perceived coercion and feelings of illness improvement [60]. Perceived coercion was not associated with dangerousness, as evaluated by professionals using standardized scales of aggression [79,101].

Multiple studies have found that lower insight is associated with higher perceived coercion [64,80,91,116,117,123,139,143]. Specifically, two studies reported that as insight improved over time, perceived coercion decreased [80,116]. One study identified a link between greater awareness of treatment efficacy and lower perceived coercion but found no association between coercion and awareness of mental illness or its social consequences [126]. Three studies reported no significant associations between insight and perceived coercion [136,150,154]. Lower global functioning was often associated with higher perceived coercion [14,20,67,74,82,94,96,116]. However, three studies found no association between the amount of coercion experienced during admission and global functioning [54,136] or impairment [101].

##### Hospitalization History and Trajectory of Care

Some studies found that a history of hospitalizations was associated with higher levels of perceived coercion [65,91,131,155,162]. However, an equal number of studies found no significant association [54,67,89,139,160], and one study reported that patients with fewer admissions perceived greater coercion [35]. Additionally, a qualitative study described the experience of returning to the hospital as feeling as though one’s entire life was placed under control [114].

Findings on the trajectory of perceived coercion over time were mixed. Some studies reported a decrease in perceived coercion between admission and discharge [94,116,124], while others found no significant change [41,43,60,65]. One study indicated that perceived coercion remained stable for 50% of patients who initially reported feeling coerced at admission [89]. In outpatient settings, however, perceived coercion tended to increase over time [73,77].

##### Type of Treatment and Quality of Care

Higher perceived coercion was associated with a negative attitude toward medication [123,139]. Individuals receiving depot medication reported greater perceived coercion compared to those taking oral medication [84]. While one study found that better medication adherence was linked to higher perceived coercion [100], others reported greater perceived coercion among nonadherent patients [56,155]. One study found that electroconvulsive therapy was not associated with high levels of perceived coercion [124]. The qualitative component of a mixed-methods study indicated that patients reported feeling coerced due to the strong emphasis placed on treatment adherence by providers, even when they personally found the medication helpful [133]. Participation in treatment planning and evaluation had only a marginal effect on perceived coercion [58]. Higher levels of perceived coercion were significantly associated with lower quality of care [159]. Similarly, being satisfied with received treatment was associated with lower perceived coercion [13,89,100,160].

Several studies examined perceived coercion among individuals receiving services from assertive community treatment teams (ACT). While two studies found no association between ACT and perceived coercion [90,92], both qualitative [83] and quantitative [73] research indicated that patients felt coerced during initial service contacts. Compared to hospital-based care or recent discharge, patients in community treatment [63] and outpatient commitment [81] reported lower levels of coercion. However, patients in outpatient commitment described phone calls and home visits as invasive and controlling [106], and one study found that individuals in assisted living were more likely to perceive coercion [98]. Women randomized to crisis house treatment experienced significantly less perceived coercion than those treated on inpatient wards [86].

#### 3.2.3. Relational

This theme focuses on the interpersonal and relational dynamics that shape individuals’ perceptions of coercion in psychiatric settings. It includes aspects such as the therapeutic relationship, the use of informal coercion, and the degree to which patients perceive procedural justice in their interactions with professionals and institutions.

##### Therapeutic Relationship

A stronger therapeutic alliance was consistently associated with lower perceived coercion [15,46,50,69,77,136]. Several studies highlighted specific relational factors that shaped patients’ perceptions of coercion. Positive influences included a collaborative and trusting relationship [83,153], encouragement from professionals [47], and their availability [83]. In one study, patients reported that nurses with more experience and greater self-confidence were less coercive [132]. Conversely, negative relational dynamics heightened perceived coercion. Patients reported not being recognized as a human and autonomous person [49,51,83,104,110,149,163], experiencing boundary violations and invasions of privacy from professionals [83], and encountering a lack of kindness (8, 41). Feelings of being ignored or unimportant were also common [49,51,89,118,145], as were perceptions of inadequate support and trust [135,145]. Some patients described interactions as authoritarian or militaristic [104] and felt that their statements were censored [152] or that they had to conform to professionals’ constructs of “adequate” behavior [163]. Perceived coercion was often linked to negative treatment by staff, particularly when professionals expressed frustration toward patients, displayed apathy, or experienced burnout [161]. The distress associated with the use of formal coercive measures was particularly intensified when patients believed decisions were made arbitrarily, punitively, or due to perceived professional incompetence [150].

##### Informal Coercion

Informal coercion involves interpersonal power dynamics between patients and professionals. Many studies have found a positive association between perceived coercion and the use of threats, leverage, or displays of force [41,45,47,48,65,85,162]. Reported threats included the possibility of outpatient commitment [65], changes in legal status [130], discharge [110], changes in medication [141], unit transfers [130], negative consequences [47], and loss of privileges [68,110,130,141]. Patients also described being pressured to take medication, accept hospital restrictions, or adhere to post-discharge treatment plans [153]. However, some studies found no association between persuasion, inducement, threats, or force and perceived coercion [82], nor between coercive strategies in outpatient settings and perceived coercion [77]. Similarly, informal coercion at admission was not linked to perceived coercion [158]. While inducement showed no significant association with perceived coercion, experiences of persuasion were associated with higher perceived coercion in one study [65]. The belief that refusing treatment would lead to involuntary admission or police intervention was also linked to greater perceived coercion [65]. In qualitative studies, patients perceived coercion when they felt that their physical integrity was compromised and that they were treated as if they had no human value [51], when they were manipulated, persuaded, blackmailed, or restricted from accessing or managing their own affairs [161], or when they were forced to accept unit transfers and medication without any legal justification [152]. Some patients reported feeling that their identity was under attack [53], while constant supervision was identified as the most negative form of informal coercion [130]. Notably, some patients perceived threats as more coercive than formal measures [130]. Being exposed to violence also contributed to perceived coercion [51].

##### Procedural Justice

Numerous quantitative studies have found an inverse relationship between levels of procedural justice and levels of perceived coercion [15,24,34,38,42,45,48,54,55,65,92,95,105,107,147,156]. Four studies specifically reported that a lack of procedural justice heightened perceived coercion even among voluntarily admitted patients [24,34,42,105]. Several qualitative studies also identified themes related to procedural justice principles (voice, respect, neutrality, trust), particularly highlighting patients’ experiences of not being heard and not receiving all necessary information. For example, greater knowledge about one’s involuntary admission was associated with lower levels of perceived coercion [147]; however, another study found that, despite being provided with information and options, patients felt pressured into hospitalization due to insufficient time for informed decision-making [89]. Some patients reported receiving unclear [163] or manipulated [161] information, leading to feelings of loss of control and objectification [153]. In a review of the literature on patients’ perspectives of electroconvulsive therapy, it was documented that only 50% of patients felt they were given sufficient information, and a third felt they did not consent freely to receiving electroconvulsive therapy despite having signed the consent form, indicating that lack of information led to feeling coerced [62]. Studies across various care settings underscore the significance of being heard. For example, not being heard by healthcare professionals increased feelings of loss of control among women hospitalized in psychiatry [141]. In assertive community treatment, patients described a lack of listening, which heightened their sensitivity to power dynamics [53]. Similarly, two studies on forced community treatment (OPC and ACT) found that not being involved in decision-making and knowing that others were making choices on their behalf was particularly distressing and intrusive [83,106]. Additional concerns included inadequate information about treatment and rights, lack of involvement in decision-making, and limited choices and options [49,89,149,153,161]. Conversely, factors that mitigated perceptions of coercion included being treated as an equal human being [33,104], perceiving professionals as well-qualified [33,49], and trusting that professionals had just intentions [33].

#### 3.2.4. Legal

This theme examines how legal frameworks and practices influence perceived coercion. It considers the impact of admission status, history of involuntary treatment, use of outpatient commitment, and other legally sanctioned measures such as seclusion and restraint. These legal dimensions often intersect with questions of capacity, autonomy, and human rights.

##### Legal Status at Admission

Numerous studies have shown that involuntary hospitalization is associated with higher perceived coercion [10,13,14,15,35,37,38,42,44,45,48,59,64,72,94,96,120,129,131,137,139,151,155,160]. Qualitative research further highlights the coercive nature of involuntary hospitalization, with individuals reporting a loss of rights [143], freedom [118], choice [51], and control over their lives [152], as well as feeling forced to accept hospitalization [104] and medication [118]. However, some studies found no significant association between perceived coercion and legal status at admission [54,89,156] or reported no differences between voluntarily and involuntarily admitted patients [55,99]. Notably, many voluntary patients also reported feeling coerced [15,35,44,54,60,64,93,105,115], with one study indicating that more voluntary patients (89%) than involuntary patients (21%) perceived coercion [93]. Additionally, patients with a history of involuntary admission reported higher perceived coercion [14,74,75,81,89,98,131], although one study found that perceived coercion decreased in individuals with a forensic history [112]. Patients subjected to outpatient commitment were more likely to perceive coercion [24,57,65,92,95,107], though two studies found no difference [56,69]. In a qualitative study, patients compared supervised discharge orders to an instrument of control [52]. Regarding decision-making capacity, three studies found that a lack of capacity to consent was associated with higher perceived coercion [79,101,113]. Additionally, one study reported that patients who consented to treatment scored higher on competence assessments and experienced lower levels of perceived coercion [113].

##### Other Restrictive Practices

Patients who experienced coercive measures such as physical restraint [111,124,127], mechanical restraint [150], chemical restraint [124], and seclusion [58,124] were more likely to perceive coercion. Seclusion and restraints were reported as highly distressing, with patients likening them to hell or prison and stating they worsened their condition [81]. However, two studies found no association between seclusion or restraint and perceived coercion at admission [137,151], and another found no significant difference in perceived coercion between individuals who had experienced at least one coercive measure and those who had not [139]. One study reported that forensic and general psychiatric admissions had similar levels of perceived coercion, despite significantly more coercive events in forensic settings [55]. Another study suggested that prior coercive experiences were linked to lower perceived coercion scores [112]. Police transportation was also perceived as highly coercive and traumatizing [121,155].

#### 3.2.5. Structural

This theme addresses structural and institutional features of psychiatric care that contribute to perceived coercion. It includes ward design, institutional rules, and the overall environment and organization of care settings. These structural factors reflect broader systemic norms and values that can profoundly affect patients’ sense of autonomy, safety, and dignity.

##### Institutional Norms and Practices

While locked wards were experienced as controlling [51,76] and were significantly associated with higher perceived coercion compared to open-door policy wards [157], it was the rigid rules and routines that were most consistently reported as restrictive in the literature [51,89,102,104,114,141,142,145,149,152,153,161]. Patients described restrictions such as prohibitions on smoking, coffee, or going outside [102,142], the inability to use a phone [152], and needing staff permission for basic needs [114,141] as profound losses of control. In both forensic and general psychiatric settings, rules were often perceived as arbitrary, leading to rights deprivation, loss of liberty, and increased uncertainty and tension with healthcare professionals [153]. The experience was particularly distressing when patients were not informed of the rules yet penalized for breaking them [89,141] or when staff enforced policies rigidly rather than flexibly [104,153,161]. Certain enforced rules were especially dehumanizing, such as requiring patients to undress, undergo searches, and wear hospital gowns in front of staff [149]—an experience that was particularly traumatic for individuals with a history of sexual abuse [114,145]. Additionally, one study reported that patients felt pressured to sign a contract upon arrival at the hospital, which led to the imposition of multiple restrictive practices and pressure to take unwanted medication [110].

##### Institutional Environment and Organization

Harmful environments that lacked therapeutic value, compromised privacy, instilled fear of other patients, and isolated individuals from their families were documented [89]. Psychiatric wards were frequently described as carceral [104,153,161] and overly medicalized, contributing to feelings of inferiority [104]. Organizational shortcomings, such as limited resources, inadequate equipment, and poor bathroom conditions, further shaped negative experiences [149]. Many patients also reported feelings of not belonging or being out of place within these settings [149,153].

## 4. Discussion

This scoping review provides a comprehensive synthesis of 143 publications on the factors associated with perceived coercion in psychiatric settings. The findings reveal that perceived coercion is a multidimensional construct shaped by a complex interplay of individual, clinical, relational, legal, and structural factors. In this discussion, we interpret the main findings in light of previous research, highlight several critical gaps and inconsistencies in the literature, and consider implications for future studies, clinical practice, and policies.

### 4.1. Main Findings

Across diverse geographic and care settings, relational and legal factors emerged as the most consistently associated with perceptions of coercion. Relational factors largely pertain to the interactions between individuals and mental health professionals. In line with previous literature, our review reinforces that the quality of the therapeutic relationship is central to how psychiatric care is experienced [164,165]. While earlier studies have associated therapeutic alliance with increased satisfaction, treatment adherence, well-being, and self-efficacy [166,167,168], this review demonstrates that a positive therapeutic relationship can also mitigate perceptions of coercion.

Studies focusing on procedural justice and informal coercion further emphasized key relational dimensions that either contribute to or alleviate feelings of coercion. These include transparent and bidirectional communication and information sharing, trust in the professionals’ intentions and competence, and the avoidance of interventions and approaches based on threats, manipulation, or other forms of informal coercion [147,150,153,161]. Though valuable, the application of procedural justice principles in practice should not be seen as a miracle solution, as their use may remain limited in terms of authenticity and organic integration [169]. As Faissner et al. (2025) note, the testimonies of experiences of coercion by patients often fail to have an impact on the professionals’ narrow view of coercion, a dynamic that points to the persistent presence of epistemic injustice and structural oppression within psychiatry [170].

Legal factors identified in the review also underline the relational nature of perceived coercion. Consistent with prior findings, our review confirms that while perceived coercion is frequently associated with involuntary treatment, it is not limited to it (réf études initiales CP). Many voluntarily admitted individuals reported high levels of coercion, often linked to subtle forms of pressure, lack of adequate information, or exclusion from meaningful decision-making processes. The use of restrictive practices such as seclusion and restraints was also associated with perceived coercion in our review, echoing long-standing concerns in the literature about their ethical and psychological impacts, as well as ongoing debates surrounding the justification of their very existence [171].

In contrast, individual characteristics (such as age, gender, and education) and clinical variables (such as diagnosis and symptom severity) yielded inconsistent associations with perceived coercion, suggesting that these factors may interact with broader systemic and interpersonal contexts rather than function as primary determinants on their own. Clinical indicators in particular provided limited insight into the lived experiences of coercion and their practical implications. While individuals with more severe symptoms were sometimes more likely to report coercion, it remains unclear whether this is due to the symptoms themselves, the higher likelihood of being subjected to formal coercive measures, or to how professionals respond to those perceived as severely ill. Literature on paternalism in psychiatric care suggests that professionals often curtail patients’ autonomy under the guise of protection, benevolence, and normative ideals of functioning [172,173,174]. Considering that individuals exhibiting more severe symptoms are more likely to be perceived as incapable of understanding and therefore of making decisions [175,176], it is not surprising that our results point to a greater perception of coercion among these individuals. A study by Sjöstrand et al. (2015) found that professionals who perceived patients as making unwise treatment decisions were more likely to judge them incapable of decision-making [177]. Yet, the literature tends to show that the majority of individuals diagnosed with severe mental health problems are capable of making decisions regarding their health [178]. This also raises questions about how severity is conceptualized and measured, typically through DSM-5-based scales that overlook broader functional and socio-structural dimensions [179]. In this regard, future research on perceived coercion and its clinical implications for professionals working with individuals from diverse backgrounds should pay close attention to how racial, ethnic, and gendered factors can influence symptom expression as well as preferences for care and treatment [180].

Another finding of interest in our review is the role of structural factors. It appears that the experience of individuals receiving psychiatric care is heavily shaped by elements such as rigid routines and strict rules that limit their autonomy and infringe upon certain rights. Moreover, much like the elements related to the quality of the therapeutic relationship and interactions with healthcare professionals, these professionals are often the ones enforcing the rules and thus perceived as the ones responsible for the negative experience. Although each professional’s approach does influence how care is perceived and the extent to which it is experienced as coercive [47,83,153], the literature also highlights that healthcare workers are trapped within systems that prevent them from intervening in ways that align with their professional values, which are centered on supporting, not controlling, the person [181,182,183]. Operating within chronically underfunded mental health systems that lack adequate resources, professionals are themselves impacted by stressed organizational environments, which undermines their ability to act outside of a paradigm focused on control, safety, and crisis management [184]. As such, research on perceived coercion should further explore the impact of public policies on mental health funding, as well as the working conditions of healthcare professionals.

### 4.2. Methodological Gaps

This scoping review, which included empirical studies, theoretical papers, reviews, and grey literature, revealed that perceived coercion in psychiatry has predominantly been studied through quantitative research methods. This dominance of quantitative methodology illustrates how studies on coercion are shaped by the biomedical model and highlights the need for more writings from individuals who identify as service users or psychiatric survivors [185]. To this end, a hierarchical approach to knowledge, where quantitative research is perceived as the highest standard, is likely to contribute to epistemic injustices. The results of this scoping review demonstrate that a broad, nuanced, and qualitative understanding of perceived coercion by people receiving psychiatric care is required [186,187].

Given the overall lack of consistent findings from quantitative studies attempting to identify significant associations between perceived coercion and various factors and considering that perceived coercion is fundamentally a subjective human experience, this methodological dominance raises important concerns as it risks simplifying a complex human experience. Notably, most studies focused on inpatient settings and relied heavily on standardized tools, such as the MacArthur Admission Experience Survey [188] or the Coercion Ladder [189], to measure coercion. While widely used and useful for comparison, these instruments offer limited and overly simplistic understanding of psychiatric coercion [190]. These scales are unable to fully capture the richness and variability of individual experiences and do not identify all the contributing elements. As such, they tend to individualize the problem, ignoring the systemic and structural dimensions of coercion.

In contrast, qualitative studies, although fewer in number, provided some of the most critical insights into the lived experience of coercion. For instance, these studies shed light on how structural factors such as rigid ward rules, locked units, lack of privacy, resource scarcity, and dehumanizing practices deeply influence individuals’ perceptions of coercion [51,89,102,104,114,141,142,145,149,152,153,161]. These findings underscore the need to shift research focus away from narrow associations with individual factors and toward structural contributors. Without disregarding the importance of continuing efforts to reduce or eliminate formal coercive practices (such as involuntary treatment and hospitalization), our review points to the need to reconceptualize coercion through a systemic lens. This includes examining how care environments, public policies, and organizational structures constrain both patient experiences and professional practices.

### 4.3. Overlooked Populations

Our review also identified other significant gaps in the literature. First, there is a near-total absence of studies considering how sexual and gender diversity shapes individuals’ pathways and experiences of coercion. Yet, recent literature on sexual and diversity minorities points to significantly higher odds of diagnosed psychiatric conditions [191,192] as well as a lack of inclusive, identity-affirming, and non-discriminative care [193]. Documenting how coercion is experienced by these persons appears fundamental to ensuring a pathway toward more inclusive psychiatric care. Additionally, very few studies addressed the role of poverty or homelessness, despite the fact that these social conditions influence mental health conditions and care access [194,195], and thus likely the experience of coercion.

Similarly, race and ethnicity have been approached in a predominantly quantitative way, often treated as a standalone variable without intersectional analysis. Most studies reviewed reported no significant association between race or ethnicity and perceived coercion. This finding may appear surprising, especially in light of well-documented evidence that Black individuals, in particular, are disproportionately subjected to coercive practices in psychiatric settings [196,197]. One study on Black persons’ experiences in psychiatric emergency departments found that they reported markedly different treatment compared to individuals who are not subject to racialization, including poorer communication and excessive use of medication [198]. Furthermore, racialized individuals are also less likely to seek care due to systemic racism in healthcare systems [199] and often have less access to voluntary services due to socioeconomic barriers [200]. Yet, studies on perceived coercion have failed to capture the lived experience of psychiatric care and its multiple dimensions. While a number of studies found that White patients reported higher levels of perceived coercion than Black patients, questions remain as to why. As Carimbocas (2023) suggests, could racialized persons be desensitized to coercion based on their repeated exposure to systemic oppression [149]? This certainly warrants further research.

In addition, a significant portion of the literature has focused on identifying sociodemographic profiles associated with higher perceived coercion, such as age or gender. However, the energy invested in determining which age group or gender is more likely to perceive coercion could arguably be more effectively directed toward identifying modifiable factors that significantly impact individuals’ lived experiences. In this regard, it is important to note that sex and gender were primarily examined quantitatively for their association with perceived coercion. While most studies did not find significant associations, ten did report higher levels of perceived coercion among women [35,36,45,71,89,94,96,99,112,137]. Still, the lack of qualitative inquiry into what it means to identify as a woman and how this may shape experiences of psychiatric care remains problematic. Biological sex tells us very little about how healthcare systems and structures could be improved to meet the specific needs of diverse populations. Literature on women’s mental health clearly points to the fact that psychiatric services have been shaped by a biomedical and androcentric vision that often fails to address the realities and needs of individuals who identify as women [201,202].

### 4.4. Strengths and Limitations of the Study

This scoping review provides a comprehensive overview of the factors studied in relation to perceived coercion in psychiatric care, offering valuable insights for both clinical practice and future research. By mapping the breadth of existing literature across individual, clinical, relational, legal, and structural dimensions, it highlights key modifiable factors, such as the therapeutic relationship, and underscores the need to consider broader systemic contributors, including institutional structures and mental health care policies. These findings can help clinicians tailor interventions to reduce perceived coercion and assist policymakers in addressing underlying systemic issues.

However, this review is not without limitations. First, we did not assess the methodological quality of the included studies. As a result, we cannot draw definitive conclusions about the strength or consistency of findings, nor can we compare results across studies. Our aim was instead to present a portrait of what has been studied to date. Second, the review was limited to publications in English and French, which may have excluded relevant studies published in other languages. Third, while some included studies referenced coercive interactions involving non-healthcare actors, such as police officers, our search strategy did not explicitly target these contexts. Yet, these interactions may contribute to a cumulative coercive experience for individuals navigating multiple institutional systems of authority, an area that warrants further investigation. In addition, the wide timespan covered by this review (1990–2025) has resulted in the inclusion of studies reflecting different cultural and temporal contexts, which may influence how coercion has been conceptualized, experienced, and reported across studies. This should be considered when interpreting the findings of this review.

Despite these limitations, this review demonstrates that perceived coercion emerges from a complex interplay of personal, relational, and structural forces, reinforcing the importance of research that interrogates not only individual-level variables but also the broader systems that shape psychiatric care.

## 5. Conclusions

This scoping review sheds light on the multifaceted nature of perceived coercion in psychiatric settings. Our findings underscore the critical role of relational dynamics between mental health professionals and the persons receiving care, but also systemic elements such as involuntary treatment frameworks, institutional norms, and resource constraints. The review also draws attention to overlooked dimensions, including the lived experiences of racialized, gender-diverse, and socioeconomically marginalized individuals, whose perspectives remain largely absent from the literature. Considering the predominance of quantitative studies, future research should prioritize qualitative inquiries that center lived experience, amplify marginalized voices, and interrogate the institutional logics that sustain coercive practices. Ultimately, addressing perceived coercion in meaningful ways requires a shift from a narrow focus on individual characteristics toward a more holistic and systemic analysis. This includes rethinking psychiatric care environments, strengthening therapeutic relationships, and reforming mental health policies to better support both care recipients and professionals.

## Figures and Tables

**Table 1:** Example of a search conducted in PUBMED.

Database	Search Using Descriptors and Keywords	Filters	Results
**PUBMED**	((“Coercion”[Mesh] OR “Involuntary Treatment, Psychiatric”[Mesh] OR “Commitment of Mentally Ill”[Mesh] OR “Restraint, Physical”[Mesh:NoExp] OR Coercion[TIAB] OR Coercing[TIAB] OR Coercive[TIAB] OR Coerced[TIAB] OR Involuntary[TIAB] OR Involuntarily[TIAB] OR Commitment[TIAB] OR commitments[TIAB] OR Restraint[TIAB] OR restrained[TIAB] OR Restraining[TIAB] OR Seclusion[TIAB] OR secluded[TIAB] OR Secluding[TIAB] OR Constraint[TIAB] OR constrained[TIAB] OR Constraining[TIAB] OR forced[TIAB] OR force[TIAB] OR compulsory[TIAB] OR intimidation[TIAB] OR intimidate[TIAB] OR intimidated[TIAB]) **AND** (“Perception”[Mesh:NoExp] OR “Social Perception”[Mesh:NoExp] OR Perception[TIAB] OR perceptions[TIAB] OR Perceived[TIAB] OR Perceive[TIAB] OR Perceiving[TIAB] OR Experience[TIAB] OR experiences[TIAB] OR Experienced[TIAB] OR Experiencing[TIAB] OR Subjective[TIAB])) **AND** (“Mental Disorders”[Mesh:NoExp] OR “Bipolar and Related Disorders”[Mesh] OR “Schizophrenia Spectrum and Other Psychotic Disorders”[Mesh] OR “Mentally Ill Persons”[Mesh] OR “Hospitals, Psychiatric”[Mesh] OR “Psychiatric Department, Hospital”[Mesh] OR Psychiatric[TIAB] OR Psychiatry[TIAB] OR “Mental health”[TIAB] OR “Mental illness”[TIAB] OR “mental illnesses”[TIAB] OR “Mentally ill”[TIAB] OR “Mental disorder”[TIAB] OR “mental disorders”[TIAB] OR “Mentally disordered”[TIAB] OR Schizophrenia[TIAB] OR Schizophrenic[TIAB] OR Psychosis[TIAB] OR Psychotic[TIAB] OR bipolar[TIAB])	English, French	4223

## Data Availability

The original contributions presented in this study are included in the article/Supplementary Materials. Further inquiries can be directed to the corresponding author.

## Supplementary Materials

The following supporting information can be downloaded at: https://www.mdpi.com/article/10.3390/healthcare13151868/s1, Table S1: PRISMA-ScR-Fillable-Checklist_ScR perceived coercion; Table S2: Grey literature search; Table S3: Perceived coercion ScR_coding and themes.

## Abbreviations

The following abbreviations are used in this manuscript:

UK	United Kingdom
USA	United States of America
