# Supplementary material for: Factors Associated with Perceived Coercion in Adults Receiving Psychiatric Care: A Scoping Review
Source: Healthcare (Basel). 2025 Jul 30;13(15):1868. doi: 10.3390/healthcare13151868 (PMC12345834; doi:10.3390/healthcare13151868)
Supplement: Supplementary file 1 [file healthcare-13-01868-s001.zip › healthcare-3752905-tableS2 (English).pdf]

Grey literature search

Perceived coercion in psychiatry

Web site consulted	Date	Retrieved documents
Google<br>www.google.ca	December 15 2022	- SORTIR À TOUT PRIX » PERSPECTIVES DES PATIENTS HOSPITALISÉS SOUS CONTRAINTE EN PSYCHIATRIE : UNE ÉTUDE PAR THÉORISATION ANCRÉE (Thèse)<br>https://serval.unil.ch/resource/serval:BIB_04D0A02A9320.P001/REF<br><br>- Perceptions and Attitudes towards Involuntary Hospital Admissions of Psychiatric Patient<br>https://prism.ucalgary.ca/bitstream/handle/1880/52081/J_Psych_Behav_Sci_Review_Perceptions%20of%20Involuntary%20Hospitalization.pdf?sequence=1&isAllowed=y<br><br>- Psychiatric Patients' experiences of Involuntary Hospital Admissions & Treatment at the Amanuel Mental Specialized Hospital : A qualitative study<br>http://etd.aau.edu.et/bitstream/handle/123456789/16256/Teferra%20Beyero%20Dr..pdf?sequence=1&isAllowed=y

		- THE USE OF COERCION IN THE FINNISH CIVIL PSYCHIATRIC INPATIENTS A PART OF THE NORDIC PROJECT PATERNALISM AND AUTONOMY http://jultika.oulu.fi/files/isbn9789514285424.pdf<br><br>- COERCION, PERCEIVED CARE AND QUALITY OF LIFE AMONG PATIENTS IN PSYCHIATRIC HOSPITALS https://www.utupub.fi/bitstream/handle/10024/94318/AnnalesD1102Soininen.pdf?sequence=2&isAllowed=y<br><br>- The Effects of Perceived Coercion on Group Attendance, Participation in Groups, and Leaving Against Medical Advice in an Inpatient Psychiatric Facility https://core.ac.uk/download/pdf/234130226.pdf
NICE https://www.nice.org.uk/	December 15 2022	No document found
European psychiatric association https://www.europsy.net/	December 15 2022	No document found
INAHTA https://www.inahta.org/	December 16 2022	No document found
American psychiatric association https://www.psychiatry.org/	December 16 2022	No document found

Royal College of Psychiatrists https://www.rcpsych.ac.uk/	December 16 2022	No document found
Theses.fr (France) http://www.theses.fr/#	December 16 2022	No document found
Érudit – theses et mémoires (Québec) https://www.erudit.org/fr/theses/	December 19 2022	No document found
DART – thèses Europe https://www.dart-europe.org/basic-search.php	December 19 2022	- Compulsory Psychiatric Care: Perspectives from the Swedish Coercion Study Patient Experiences, Documented Measures, Next of Kins' Attitudes and Outcome http://uu.diva-portal.org/smash/get/diva2:171742/FULLTEXT01.pdf<br>- On dignity in inpatient psychiatric care http://www.tara.tcd.ie/bitstream/handle/2262/97865/Plunkett_Thesis.pdf?sequence=1&isAllowed=y<br>- Patient and Staff Perceptions of Medication Administration and Locked Entrance Doors at Psychiatric Wards http://uu.diva-portal.org/smash/get/diva2:167321/FULLTEXT01.pdf<br>- Making sense of Community Treatment

		Orders: the service-user experience https://etheses.whiterose.ac.uk/19470/1/Making%20sense%20of%20CTOs%20Thesis.pdf<br><br>- Patient and staff experiences of coercive care http://wrap.warwick.ac.uk/59329/1/WRAP_THESIS_Davies_%28nee_Morgan%29_2012.pdf<br><br>- Service users' experiences of being sectioned under the Mental Health Act https://eprints.soton.ac.uk/467060/1/1117183.pdf<br><br>- PERCEIVED COERCION, ADHERENCE TO TREATMENT AND PERSONALITY TRAITS; AN OBSERVATIONAL CROSS SECTIONAL STUDY https://pure.manchester.ac.uk/ws/files/54516296/FULL_TEXT.PDF
Open access thesis and dissertations https://oatd.org/	December 19 2022	- The effects of perceived coercion and empowerment on motivational processes for adults ordered to attend substance abuse treatment: a mixed methods analysis. https://collections.lib.utah.edu/details?id=197016

		- Former Client Perspectives on Perceived Choice, Control, and Coercion in Eating Disorder Treatment. https://etd.ohiolink.edu/apexprod/rws_etd/send_file/send?accession=miami1301781198&disposition=inline<br><br>- Perceptions of coercion of patients subject to the New Zealand Mental Health (Compulsory Assessment and Treatment) Act 1992 https://researchspace.auckland.ac.nz/handle/2292/3187<br><br>- Service users' experiences of coercion and autonomy in inpatient mental health services. https://repository.canterbury.ac.uk/download/7d92e7ead64720f544f698797a0091be502ee85d68b9cd3b232dc922ac7cb3d8/1918873/Remy_Gray_MRP_2019.pdf
Grey literature report http://www.greylit.org/	December 19 2022	No document found
Association québécoise des infirmières et infirmiers en santé mentale https://aqiism.org/	December 19 2022	No document found

Association des médecins psychiatres du Québec https://ampq.org/https://ampq.org/	December 19 2022	No document found
L'Association des groupes d'intervention en défense des droits en santé mentale du Québec http://www.agidd.org/	December 19 2022	No document found
Association des psychiatres du Canada https://www.cpa-apc.org/	December 19 2022	No document found
American psychiatric nurses association https://www.apna.org/	December 19 2022	No document found

Keywords:

French

Santé mentale, psychiatrie, psychiatrique, coercition; contrainte; traitement involontaire; hospitalisation involontaire; perception; perçue

English

Mental(ly) ; psychiatry; psychiatric; coercion; coercive ; involuntary treatment; involuntary hospitaliz(s)ation ; compulsory; perceived; perception
